# Supplementary material for: A predictive computational platform for optimizing the design of bioartificial pancreas devices
Source: Nat Commun. 2022 Oct 13;13:6031. doi: 10.1038/s41467-022-33760-5 (PMC9561707; doi:10.1038/s41467-022-33760-5)
Supplement: Supplementary file 1 — Supplementary Information [file 41467_2022_33760_MOESM1_ESM.pdf]

Supplementary Information

A predictive computational platform for optimizing the design of bioartificial pancreas devices

Alexander U. Ernst[a], Long-Hai Wang[a,b]*, Scott C. Worland[a], Braulio A. Marfil-Garza[c], Xi Wang[a], Wanjun Liu[a], Alan Chiu[a], Tatsuya Kin[c,d], Doug O'Gorman[c,d], Scott Steinschneider[a], Ashim K. Datta[a], Klearchos K. Papas[e], A. M. James Shapiro[c,d], Minglin Ma[a]*

Affiliations:

[a]Biological and Environmental Engineering, Cornell University, Ithaca, NY, USA

[b]Department of Polymer Science and Engineering, University of Science and Technology of China, Hefei, Anhui, China (current address)

[c]Department of Surgery, University of Alberta, Edmonton, AB, Canada

[d]Clinical Islet Transplant Program, University of Alberta, Edmonton, AB, Canada

[e]Department of Surgery, University of Arizona, Tucson, AZ, USA

*Minglin Ma and Long-Hai Wang

Email: mm826@cornell.edu; hiwang@ustc.edu.cn

Supplementary Materials and Methods

Supplementary Tables 1–7

Supplementary Figs. 1–37

Supplementary Materials and Methods

Size distribution fitting

Size distribution fitting of human islet isolations followed the tabular optimization method[1]. 129 isolations from deceased human donors were analyzed, with a purity, as quantified by their own analysis, of 0.79 $\pm$ 0.16. In total, 55 of the isolations were from female patients and the remaining 74 were from males, 53.7 $\pm$ 14.1 years of age at the time of donation. For each isolation, the number of islets in 50 μm diameter size groups (50–100, 100–150, 150–200, 200–250, 250–300, 300–350, and 350–400 μm) were recorded in the Alberta Diabetes Institute IsletCore database at the University of Alberta. Raw counts were converted to frequency by dividing the size group count by the total count. It was assumed that the islet sizes were described by either lognormal (subscript L) or Weibull (subscript W) distributions, the corresponding probability density functions (f_L and f_W, respectively) given by

$$f_L(d) = \frac{1}{d\alpha_n\sqrt{2\pi}}\exp\left(-\frac{(\ln(d/\beta_n))^2}{2\alpha_n^2}\right) \tag{S1}$$

and

$$f_W(d) = \frac{\alpha_n}{\beta_n}\left(\frac{d}{\beta_n}\right)^{\alpha_n-1}\exp\left(-\left(\frac{d}{\beta_n}\right)^{\alpha_n}\right) \tag{S2}$$

where $\exp$ represents the exponential function. Above, d represents the islet diameter and parameters $\alpha_n > 0$ and $\beta_n > 0$ represent the shape and scale coefficients of the probability density function on a number basis, respectively. The cumulative probability densities for both distributions (F_L and F_W, respectively), given by the integral with respect to d between 0 and ∞ of f_L and f_W of Eqs. (S1) and (S2), are thus

$$F_L(d) = \frac{1}{2} + \frac{1}{2}\operatorname{erf}\left(\frac{\ln(d/\beta_n)}{\sqrt{2}\alpha_n}\right) \tag{S3}$$

and

$$F_W(d) = 1 - \exp\left(-\left(\frac{d}{\beta_n}\right)^{\alpha_n}\right) \tag{S4}$$

where erf is the error function. The frequency, P_L or P_W, of islets in any size group defined by minimum diameter d_1 and maximum diameter d_2 is therefore given, for lognormal and Weibull distribution types, respectively, by

$$P_L(d_1 < d \leq d_2) = F_L(d_2) - F_L(d_1) \tag{S5}$$

and

$$P_W(d_1 < d \leq d_2) = F_W(d_2) - F_W(d_1) \quad \text{(S6)}$$

To determine the parameters for each distribution, P_L and P_W were calculated for each size group (i.e., 50–100, 100–150 μm, etc.) for optimal α_n and β_n, calculated using Excel's Solver Tool instructed to minimize the root-mean-square error (RMSE) between the actual frequency and P_L and P_W. For the description of the species islet size distribution (Supplementary Table 4 and Fig. 1e), the frequency of the aggregate count was used. For determining human islet size distributions from individual subjects (Fig. 3b and Supplementary Fig. 12), best-fit lognormal parameters were obtained by the above method from the islet size group frequency data of each isolation independently.

The size distributions of all other islet and SC-β sources were calculated from isolation image trace data. In ImageJ, islets/SC-βs were manually traced in their images, and the area, A, was measured, and converted into effective diameter (d), by

$$d = 2\sqrt{\frac{A}{\pi}} \quad \text{(S7)}$$

The minor diameter, a, and major diameter, b, were also measured for mouse islets, juvenile porcine islets, and human islets, the latter selected from two images chosen at random of dithizone (DTZ)-stained aliquots from the Alberta Islet Core. All cell clusters in the SC-β batch images were measured, whereas in primary islet isolation images, clusters without a distinguishable peripheral border were excluded from measurement. Cell clusters partially cropped at the edge of isolation images were also excluded from measurement. Visual examples of the inclusion criteria are provided in Supplementary Fig. 3f. First, actual cumulative densities of d were computed from raw trace data (Supplementary Fig. 4, grey circles). Then, optimal α_n and β_n for lognormal and Weibull cumulative density functions (F_L and F_W, Eqs. (S3) and (S4)) were obtained by minimizing the RMSE.

For both SC-β sources, it was first assumed that the diameters were normally distributed, according to the normal probability density function f_N

$$f_N(d) = \frac{1}{\sigma\sqrt{2\pi}} \exp\left(-\frac{1}{2}\left(\frac{d-\mu}{\sigma}\right)^2\right) \quad \text{(S8)}$$

where μ is the mean diameter and σ is the standard deviation. Again, actual cumulative density of d, collected from image analyses, was fit to the cumulative density function of a normal distribution, F_N

$$F_N(d) = \frac{1}{2}\left[1 + \operatorname{erf}\left(\frac{d-\mu}{\sigma\sqrt{2}}\right)\right] \quad \text{(S9)}$$

However, NN SC-βs failed normality tests (Shapiro-Wilk). Though it was found that the normal distribution was a more robust fit for WU SC-βs (Supplementary Fig. 4f,g), the Weibull distribution was nevertheless quite a robust fit, and additionally does not define a positive probability of sampling a negative diameter value, which is obviously nonphysical. For these reasons, the Weibull function was used to describe the size distributions of both SC-β sources.

For islet sources described best by lognormal distributions (rat, juvenile porcine, and human islets), the parameters of the volume-weighted size distribution, α_v and β_v, were calculated by the following conversions:

$$\alpha_v = \alpha_n \tag{S10}$$

and

$$\beta_v = \beta_n \exp(3\alpha_n^2) \tag{S11}$$

Volume-weighted parameters α_v and β_v for the remaining cell sources described best by Weibull distributions (mouse islets, NN SC-βs, and WU SC-βs) were obtained numerically. That is, the actual cumulative frequency of the islet/SC-β volumes (V_c) assuming spherical cell clusters ($V_c = \pi d^3/6$) was computed, and then optimal α_v and β_v of the best-fit F_W or F_N were obtained by minimizing the RMSE as previously.

Mean (unadjusted and volume-weighted) diameters ($\bar{d}_n$ and $\bar{d}_v$), given in Supplementary Table 4, were calculated, with the appropriate substitutions for α and β, according to below:

(Lognormal) $$\bar{d} = \beta\left[\exp\left(\frac{\alpha^2}{2}\right)\right] \tag{S12}$$

and

(Weibull) $$\bar{d} = \beta\Gamma\left(1 + \frac{1}{\alpha}\right) \tag{S13}$$

where Γ represents the gamma function.

Hierarchical probabilistic Monte Carlo modeling for simulating human islet size distributions

A three-level hierarchical probabilistic Monte Carlo model (Supplementary Fig. 12e) was employed to simulate human islet distributions described by the lognormal distribution with parameters $\alpha_n \sim N(\mu_\alpha, \sigma_\alpha)$ and $\beta_n \sim N(\mu_\beta, \sigma_\beta)$. A Shapiro-Wilk test found that α_n and β_n were normally distributed and a Pearson's correlation test found that they were also uncorrelated (Supplementary Fig. 12b–d), thus each parameter was considered independently. We assumed that each hyperparameter was also normally distributed:

$\mu_\alpha \sim N(\mu_{\mu_\alpha}, \sigma_{\mu_\alpha})$, $\sigma_\alpha \sim N(\mu_{\sigma_\alpha}, \sigma_{\sigma_\alpha})$, and $\mu_\beta \sim N\left(\mu_{\mu_\beta}, \sigma_{\mu_\beta}\right)$, $\sigma_\beta \sim N\left(\mu_{\sigma_\beta}, \sigma_{\sigma_\beta}\right)$. Hyperparameter values are given in Supplementary Fig. 12g.

General calculations

Calculations of islet/SC-β outcomes were performed *ex post facto* (i.e., after the solution of the internal oxygen distribution had been obtained). The mean pO_2 of the cell cluster, $\bar{p}_c$, was calculated as

$$\bar{p}_c = \frac{1}{V_c} \iiint_C p \, dV \tag{S14}$$

where V_c represents the volume of the islet/SC-β cell cluster, and the integration domain, C, represents the region of the cell cluster.

The oxygen-dependent insulin secretion potential (Supplementary Fig. 9c), S, was defined only in the cell clusters and modeled according to the Hill relationship[2-4], in favor of the bilinear[5,6] or polynomial[7] formulations used by others, based on studies of isolated islets exposed to variable media oxygen levels[8]

$$S = \begin{cases} 0, & p < p_N \\ \dfrac{p^{n_S}}{p^{n_S} + (K_S)^{n_S}}, & p \geq p_N \end{cases} \tag{S15}$$

with half-maximal coefficient K_S (in mmHg) and Hill coefficient n_S (unitless). The step-down function again represents the loss of insulin secretion capacity of necrotic cells. More precisely, S defines the relative second-phase insulin secretion rate, rather than insulin concentration, and is hence not defined in the hydrogel. Accordingly, the volume-average insulin secretion potential of the cluster, $\bar{S}_c$, was given by

$$\bar{S}_c = \frac{1}{V_c} \iiint_C S \, dV \tag{S16}$$

We defined the loss of insulin secretion potential of the cell cluster, Ψ_c, simply as

$$\Psi_c = 1 - \bar{S}_c \tag{S17}$$

The necrotic fraction of the cell cluster, N_c, was defined as the volume of tissue for which $p < p_N$, relative to the total cell cluster volume:

$$N_c = \frac{1}{V_c} \iiint_{C,\ p<p_N} dV \tag{S18}$$

Finally, the average values for the entire encapsulated islet or SC-β population, given by $\bar{p}$, Ψ, and N, were calculated by the volume-weighted average over all n_c cell clusters

$$\bar{p} = \sum_{c=1}^{n_c} V_c \bar{p}_c \bigg/ \sum_{c=1}^{n_c} V_c \tag{S19}$$

$$\Psi = \sum_{c=1}^{n_c} V_c \Psi_c \bigg/ \sum_{c=1}^{n_c} V_c \tag{S20}$$

and

$$N = \sum_{c=1}^{n_c} V_c N_c \bigg/ \sum_{c=1}^{n_c} V_c \tag{S21}$$

The nominal islet equivalent volume (IEQ) of a cell cluster is defined as $(\mathrm{IEQ})_c = V_c / V_{1\mathrm{IEQ}}$ where $V_{1\mathrm{IEQ}}$ = 1.77 × 10^{-12} m^3 represents the volume of a standard islet (a sphere with a diameter of 150 μm) [9]. We defined the functional IEQ, $(\mathrm{fIEQ})_c$, as the nominal IEQ multiplied by its insulin secretion potential:

$$(\mathrm{fIEQ})_c = (\mathrm{IEQ})_c \cdot \bar{S}_c \tag{S22}$$

The size group (e.g., between minimum diameter d_1 and maximum diameter d_2)-to-fIEQ conversion coefficients, $\kappa_{\mathrm{fIEQ}|d_1 d_2}$, were given by the volume-weighted average of the $(\mathrm{fIEQ})_c$ values of the clusters within the size group:

$$\kappa_{\mathrm{fIEQ}|d_1 d_2} = \frac{1}{n_{d_1 d_2} V_{1\mathrm{IEQ}}} \left(\sum_{c=1}^{n_{d_1 d_2}} V_c \bar{S}_c \right) \tag{S23}$$

where $n_{d_1 d_2}$ represents the number of simulated cell clusters in size group d_1-to-d_2.

Optimization calculations

The curative dose for an average patient being defined as 500 k fIEQ was decided upon by the following reasoning. It has been shown that doses of approximately 13 k IEQ per kg body weight of a human recipient are most often required to achieve insulin independence in the intraportal transplantation model[10]. However, a variable but significant proportion (~60%) of the islet mass fails to become engrafted in the first few days following delivery due to several factors[11,12]. We assume that the portion of islets which survive become vascularized and are therefore functionally unimpaired, and consider a patient of average male weight of 90 kg, and an engraftment rate of 0.4, and thus arrive at a curative cell dose ($\mathrm{fIEQ}_{\mathrm{cure}}$) of

$$\mathrm{fIEQ}_{\mathrm{cure}} = \frac{13{,}000\ \mathrm{IEQ}}{\mathrm{kg\ body\ weight}} \times \frac{90\ \mathrm{kg\ body\ weight}}{\mathrm{patient}} \times \frac{1\ \mathrm{fIEQ}}{1\ \mathrm{IEQ}} \times 0.4 = \frac{468{,}000\ \mathrm{fIEQ}}{\mathrm{patient}} \tag{S24}$$

which is rounded to 500 k fIEQ.

The total size and volume of a device required to deliver 500 k fIEQ was calculated as follows. First, we may compute the marginal fIEQ per unit volume of device (V_d), m_V

$$m_V = \frac{\text{fIEQ}}{V_d} \tag{S25}$$

The net volume of the cell cluster population (V_p) in the planar slab and cylindrical structure is given by its volume fraction within the device

$$V_p = \rho V_d \tag{S26}$$

where ρ represents the cell density as a fraction of the total device volume. Furthermore, the fIEQ is given by the islet volume, in IEQ, adjusted by the fractional functional capacity (Eq. (3)) and can therefore be rewritten, in terms of the device volume (V_d) and fractional loss of insulin secretory potential (Ψ) as

$$\text{fIEQ} = \frac{\rho V_d}{V_{1\text{IEQ}}}(1 - \Psi) \tag{S27}$$

Substituting this relationship into Eq. (S25) yields

$$m_V = \frac{\rho(1 - \Psi)}{V_{1IEQ}} \tag{S28}$$

For the hollow cylinder system, the volume of the acellular core must be accounted for. The total device volume of this system is

$$V_d = \pi(r_i + \tau)^2 L_d \tag{S29}$$

where r_i represents the passive core radius, τ the thickness of the cell layer, and L_d the device length. The cell cluster volume for this system is given by the volume fraction in the cell layer:

$$V_p = \rho(\pi((r_i + \tau)^2 - r_i^2)L_d) \tag{S30}$$

Thus, m_v for the hollow cylinder geometry is given by

$$m_V = \frac{\rho(1 - \Psi)}{V_{1IEQ}}\left(1 - \frac{r_i^2}{(r_i + \tau)^2}\right) \tag{S31}$$

following the appropriate substitutions to Eq. (S25).

Given the marginal fIEQ per unit volume (m_v), we can derive the curative volume (V_{cure}) of a device containing a curative dose ($\text{fIEQ}_{\text{cure}}$) for all geometries:

$$V_{\text{cure}} = \frac{\text{fIEQ}_{\text{cure}}}{m_v} \tag{S32}$$

We may express V_{cure} in terms of the volumetric cell density (ρ) and the fractional loss of function (Ψ) by the substitution of Eq. (S27) into Eq. (S32), for the planar slab and cylindrical geometries as

$$V_{\text{cure}} = \left(\frac{V_{1\text{IEQ}}}{\rho(1-\Psi)}\right) \text{fIEQ}_{\text{cure}} \tag{S33}$$

and for the hollow cylinder geometry as

$$L_{\text{cure}} = \left(\frac{V_{1\text{IEQ}}}{\rho(1-\Psi)\left(1-\frac{r_i^2}{(r_i+\tau)^2}\right)}\right) \text{fIEQ}_{\text{cure}} \tag{S34}$$

By extension, using the geometrical relationship between the volume and diameter of a cylinder, we can find the diameter of a device (D_{cure}) containing a curative islet payload for the planar slab:

$$D_{\text{cure}} = 2\sqrt{\frac{V_{\text{cure}}}{\pi\tau}} \tag{S35}$$

where τ represents the thickness of the planar slab. Likewise, the curative length (L_{cure}) of the cylinder is

$$L_{\text{cure}} = \frac{V_{\text{cure}}}{\pi r^2} \tag{S36}$$

where r represents the radius of the cylinder. Finally, L_{cure} for the hollow cylinder is

$$L_{\text{cure}} = \frac{V_{\text{cure}}}{\pi(r_i+\tau)^2} \tag{S37}$$

For completeness, we may express the characteristic curative sizes in terms of the original inputs via the appropriate substitutions. For the planar slab, D_{cure} is given by

$$D_{\text{cure}} = \sqrt{\frac{V_{1\text{IEQ}} \cdot \text{fIEQ}_{\text{cure}}}{\pi\tau\rho(1-\Psi)}} \tag{S38}$$

for the cylinder, L_{cure} is given by

$$L_{\text{cure}} = \frac{V_{1\text{IEQ}} \cdot \text{fIEQ}_{\text{cure}}}{\pi r^2 \rho(1-\Psi)} \tag{S39}$$

and for the hollow cylinder, L_{cure} is given by

$$L_{\text{cure}} = \frac{V_{1\text{IEQ}} \cdot \text{fIEQ}_{\text{cure}}}{\pi(1-\Psi)\left(1-\frac{r_i^2}{(r_i+\tau)^2}\right)(\pi(r_i+\tau)^2)} \tag{S40a}$$

which may be simplified to

$$L_{\text{cure}} = \frac{V_{1\text{IEQ}} \cdot \text{fIEQ}_{\text{cure}}}{\pi\tau\rho(1-\Psi)(2r_i+\tau)} \tag{S40b}$$

Lastly, we may calculate the actual volume of islets required to achieve insulin independence (IEQ_{cure}) as the curative dose divided by the fractional insulin secretion potential

$$\text{IEQ}_{\text{cure}} = \frac{\text{fIEQ}_{\text{cure}}}{1-\Psi} \tag{S41}$$

Each input variable combination of the optimization studies (Fig. 5) was run in triplicate, with each iteration simulating 750 IEQ (except for studies of the cylinder, in which 500 IEQ were simulated). Diameter selections and seeding positions were randomized each iteration.

Model implementation

Parameter values (Supplementary Table 7) were obtained from the literature. A variety of hydrogels have been employed for islet encapsulation[13,14], thus it was necessary to select one representative material. Because of its widespread use both presently and historically, we selected 2% (w/v) alginate as this model material and obtained parameters for the hydrogel from investigations of this material. The partial pressure-dependent solubility coefficient at 37ºC of oxygen in hydrogel ($\alpha_{O_2,h}$) was implemented as 1.24×10^{-3} mol m^{-3} $mmHg^{-1}$, which is 98% that of water[6,15]. The diffusion coefficient at 37ºC of oxygen in hydrogel ($D_{O_2,h}$), obtained from reported measurements[15,16], was implemented as 2.70×10^{-9} m^2 s^{-1}. The selection of these values, were also, in part, following literature consensus[3,7,15]. For simulations of the model BAP device (Fig. 4f–h and Supplementary Fig. 17b–f), the nylon suture thread was modeled as a bulk nylon material with a partial pressure-dependent solubility coefficient of oxygen of 1.90×10^{-3} mol m^{-3} $mmHg^{-1}$ and an oxygen diffusion coefficient of 5.00×10^{-13} m^2 s^{-1}, both at 37ºC[17].

We assumed that all physical parameters in the cell clusters were independent of the cell source. Following previous use[6,15], the partial pressure-dependent solubility coefficient at 37ºC of oxygen in the cell clusters ($\alpha_{O_2,c}$) was implemented as 1.02×10^{-3} mol m^{-3} $mmHg^{-1}$. A diffusion coefficient at 37ºC of oxygen in the cell clusters (D_{O_2c}) of 2.00×10^{-9} m^2 s^{-1} was used, also following previous reports[2-4]. Likewise, all parameters relating to oxygen consumption and insulin secretion potential were considered independent of the cell source, except for the maximum basal OCRs (V_{max}), which were obtained from the references provided in Supplementary Table 5. The Michaelis-Menten half-maximal coefficient of oxygen consumption (K_V = 0.80 mmHg), half-maximal coefficient of oxygen-dependent insulin secretion capacity (K_S = 2.00 mmHg), Hill coefficient of oxygen-dependent insulin secretion (n_S = 3), and necrotic threshold (p_N = 0.08 mmHg), all followed the derivations for a similar problem described elsewhere[2-4].

The default constant external pO_2 value, p_{ext}, was implemented as 40 mmHg. There is considerable variability in reported pO_2 levels in common islet delivery device transplantation sites (Supplementary Table 1), ranging from < 20 to > 60 mmHg. We note that the oxygen tension on the surface of a device may be lower than that of the surrounding environment as a gradient may be present in the surrounding tissue and the deposition of the fibrotic layer may present an additional layer of oxygen transfer resistance[18,19], but it also may be higher if vascularization occurs near or upon the surface of the graft. Furthermore, the oxygen environment in one subject may change throughout the day, especially in the subcutaneous space, in response to behavioral factors such as physical compression or exercise[20]. Given this uncertainty, we therefore considered $p_{\text{ext}} = 40$ mmHg as a justifiable default value, with an exploration of the model sensitivity to p_{ext} provided in Supplementary Fig. 36.

The mathematical model (Eqs. (7) and (8), with the relevant boundary conditions) were solved using the finite element computational software COMSOL Multiphysics with MATLAB, a software package that includes wrapper functions to implement COMSOL model development within MATLAB's coding environment. The use of MATLAB allowed for the simple application of stochastic methods (needed for building the 3D model geometry) to be combined with COMSOL's powerful finite element solver. An array of sphere diameters, selected according to their probability densities (of Supplementary Table 4), up to the desired cell cluster volume was created. Then, a seeding algorithm positioned the spheres according to a uniform distribution in all spatial directions within the region of the device, while repositioning spheres which overlapped with any other. For every simulation, a custom mesh was generating using COMSOL's "Free Tetrahedral" option, with a maximum element size of 2.5×10^{-3} m, a minimum element size of 4×10^{-7} m, a curvature factor of 0.26 (unitless), a "resolution of narrow regions" parameter of 2.5 (unitless), and a maximum element growth rate of 1.13 (unitless). It was ensured that the solutions were mesh independent (Supplementary Fig. 9d,e).

Machine learning model development

Training data. The training data for the models consisted of 626,751 κ_{fIEQ} values generated using SHARP, a combination of 85 volumetric cell densities, ρ, and geometry variables (referring to the planar slab thickness τ, cylinder diameter D, or hollow cylinder inner diameter D_i, hereafter generalized for simplicity as Ω) generated using an evenly spaced grid, and ~2,500 diameters for each combination sampled the human islet diameter distribution described in Supplementary Table 4. The training grid and diameter counts are provided in Supplementary Data 1. The training data was then summarized using the average $\bar{S} = (1 - \Psi)$ values for each combination of ρ, Ω, and the conventional diameter bins (i.e., 0–50, 50–100, 100–50 µm, etc.). This resulted in 2,010 training observations which are provided in Supplementary Data 2 and Supplementary Fig. 30. We explored the accuracy and generalizability (i.e., the "performance") of prediction models trained using the raw data (n = 626,751) versus the summarized data (n = 2,010) and observed that the performance was equal between the two and that models trained using the summarized data resulted in a 10-fold increase in inference speed. Therefore, we used the summarized data. The

models were trained using $\bar{S}$ as the target variable and ρ, Ω, and the average diameter for each diameter bin as the predictor variables.

Hyperparameter tuning. A hyperparameter is any free parameter in a model that is provided by the user and must be learned through a process of searching for values of model parameters that optimize a predefined loss function (e.g., the RMSE). The goal is to find hyperparameters that generalize well when making predictions on new data. We used 10-fold cross validation and a 100-point grid to select the final hyperparameters for each model. The grid was generated using a maximum entropy design[21] that maintains greater coverage of the candidate parameter space than uniform random sampling while requiring fewer sampling points than an evenly spaced grid. The models were trained for each cross-validation fold and for each combination of hyperparameter values. The final hyperparameters for each model and geometry are provided in Supplementary Data 3.

Out-of-sample predictions, model ensemble. The optimal hyperparameters obtained during the tuning process were then used to make out-of-sample predictions using the cross-validation folds. For each geometry and model, the model was fit to the 90% of the data (10–1 folds) and used to estimate the remaining 10%. This was repeated until every data point in the training data had been estimated out-of-sample, i.e., was estimated using a model that was not fit on the data being estimated (Supplementary Data 4). The hyperparameter tuning function is provided in Supplementary Data 5. The ρ and Ω values were used to generate "low" ($\Omega \leq$ median Ω value for geometry and $\rho \leq$ median ρ value for geometry) and "high" bins ($\Omega >$ median Ω value for geometry and $\rho >$ median ρ value for geometry) which were then used, along with the geometries and the diameter bins, to group the estimated and actual values of $\bar{S}$. The Pearson correlation coefficient was estimated for each of these groups to generate model weights (Supplementary Fig. 32) for the final ensemble model. The six machine learning models described in Fig. 6a (middle panel) were used to make predictions for $\bar{S}$ using the covariates, and the predictions from each model are combined using a weighted average where the weights are derived from the Pearson correlation coefficient described above. Final model specifications are provided in Supplementary Data 5.

Software. The model training pipeline was built using the tidymodel[22] framework in R statistical programming language.

Supplementary Tables

Supplementary Table 1 Oxygen availability in extravascular BAP transplantation sites. Literature-reported measurements of the pO_2 (in mmHg) in common transplantation sites suitable for BAP transplantation. The animal model, measurement method, and reported pO_2 are presented.

Animal model	Measurement method	pO_2 (mmHg)	SI reference
Subcutaneous			
Human	Clark-type microelectrode	35 ± 8	23
Nonhuman primate	Clark-type microelectrode	36 ± 6	24
Pig	Clark-type microelectrode	58 ± 16	25
Rat	Electron paramagnetic resonance imaging	29–38	26
Rat	Electron paramagnetic resonance imaging	34–40	27
Rat	Optical microelectrode	49 ± 9	28
Rat	Optical microelectrode	22–30	19
Rat	^{19}F nuclear magnetic resonance imaging	8–14	19
Intraperitoneal			
Nonhuman primate	Clark-type microelectrode	31 ± 7	24
Rat	Clark-type microelectrode	53–58	29
Rat	^{19}F nuclear magnetic resonance imaging	52 ± 7	30
Rat	^{19}F nuclear magnetic resonance imaging	21 ± 13	31
Mouse	Clark-type microelectrode	45 ± 15	32

Data are presented as range or mean ± SD.

Supplementary Table 2 Size distribution raw data. Sample, number of islets (n), mean number-basis diameter ($\bar{d}_n$) and maximum diameter are shown for each cell source.

Cell source	Sample	n	$\bar{d}_n$ (μm)	$d_{\max}$ (μm)
Mouse islets	Batch 1	750	158.5	355.8
	Batch 2	818	147.7	305.2
	Aggregate	1568	152.0	355.8
Rat islets	Batch 1	562	91.0	233.1
	Batch 2	1204	114.3	281.8
	Batch 3	452	122.3	282.9
	Batch 4	646	126.8	317.4
	Batch 5	198	218.7	380.8
	Aggregate	3062	122.0	380.8
Juvenile porcine islets	Batch 1	493	165.7	604.7
	Batch 2	2555	181.7	530.6
	Aggregate	3048	179.4	604.7
Human islets	Aggregate	20432	123.2	>400
NN SC-βs	Batch 1	446	143.4	227.6
	Batch 2	97	171.5	223.2
	Batch 3	59	148.7	184.8
	Aggregate	602	148.3	227.6
WU SC-βs	Batch 1	46	247.9	331.8
	Batch 2	38	269.2	327.0
	Batch 3	61	221.5	296.1
	Aggregate	145	240.0	327.0

Supplementary Table 3 Human islet distribution curve fitting. Observed frequency was collected from aggregated data. The lognormal distribution was a better fit according to the root-mean-square error (RMSE).

Size group (μm)	Observed frequency	Best-fit Weibull distribution-predicted frequency	Best-fit lognormal distribution-predicted frequency
< 50	-	0.0662	0.0104
51–100	0.3319	0.3291	0.3339
101–150	0.4095	0.3993	0.4096
151–200	0.1537	0.1639	0.1645
201–250	0.0587	0.0207	0.0462
251–300	0.0253	0.0007	0.0117
301–350	0.0132	0.0000	0.0029
351–400	0.0078	0.0000	0.0008
	**RMSE:**	0.0189	0.0094

Supplementary Table 4 Islet and SC-β size distribution properties. Best-fit distribution parameters (α and β) and mean diameter (d) on a number and volume basis (subscripts n and v, respectively) determined for each cell source. Mean diameters provided only next to the distribution type determined to be the best fit, according to the RMSE.

	Best-fit distribution parameters					
**Cell source**	α_n	β_n	α_v	β_v	$\bar{d}_n$ (μm)	$\bar{d}_v$ (μm)
Best-fit lognormal parameters						
Rat islets‡	0.40	112.6	0.40	182.0	122.0	197.1
Juvenile porcine islets‡	0.32	152.2	0.32	207.2	179.4	218.1
Human islets‡	0.36	115.4	0.36	170.2	123.2	181.6
Mouse islets	0.30	149.7	0.30	196.1		
NN SC-βs	0.14	149.5	0.14	158.6		
MM SC-βs	0.14	241.7	0.14	256.3		
Best-fit Weibull parameters						
Rat islets	2.78	131.5	3.48	228.4		
Juvenile porcine islets	3.53	193.4	4.18	254.8		
Human islets	2.89	126.3	2.39	242.0		
Mouse islets‡	3.67	168.5	5.00	206.3	152.0	189.4
NN SC-βs‡	7.87	157.6	9.29	166.3	148.3	157.7
MM SC-βs‡	8.14	254.7	9.58	267.3	240.0	253.8

†Probability density functions of the lognormal and Weibull distributions are given by Eqs. (S1) and (S2); cumulative probability density functions are given by Eqs. (S3) and (S4).

‡Symbol indicates best-fit distribution type for that species, defined by lower RMSE.

Supplementary Table 5 Reported islet/SC-β basal oxygen consumption rates (OCRs). Maximum basal OCRs (V_{max}) reported for cell sources, shown on a DNA-normalized basis and on a volumetric basis. Units were converted by the standard[33] of 10.4 ng DNA IEQ^{-1}.

**Islet source**	**Basal maximum OCR (V_{max})**		**SI reference**
	**(nmol O_2 min^{-1} mg DNA^{-1})**	**(mol O_2 m^{-3} s^{-1})**	
Mouse islets	300.0	0.0294	34
Rat islets	346.6	0.0340	35
Juvenile porcine islets	175.0	0.0172	36
Human islets	136.6	0.0134	37
NN SC-βs	225.9	0.0222	Personal communication

Supplementary Table 6 Dependence of functional outcomes on islet size distributions. Pearson's correlation coefficient (r) for various distribution properties versus percent loss of insulin secretory function (Ψ) in the hypothetical construct containing human islets with size distributions simulated by the hierarchical probabilistic Monte Carlo model (Fig. 3).

**Lognormal distribution parameter**	**Pearson's r, distribution parameter versus Ψ**
Shape parameter[†], α	0.81
Number distribution scale parameter, β_n	0.47
Volume distribution scale parameter, β_v	0.93
Number-mean diameter, $\bar{d}_n$	0.64
Volume-mean diameter, $\bar{d}_v$	0.93
Number distribution standard deviation, SD_n	0.95
Volume distribution standard deviation, SD_v	0.87
Tail weight index[‡], TWI	0.81
Islet size index, ISI	0.86

[†]Shape parameter (α) is the same for both number- and volume-basis distributions (Eq. (S10)).

[‡]$\mathrm{TWI} = \left(\frac{F^{-1}(0.99)-F^{-1}(0.5)}{F^{-1}(0.75)-F^{-1}(0.5)}\right)\Big/\left(\frac{\Phi^{-1}(0.99)-\Phi^{-1}(0.5)}{\Phi^{-1}(0.75)-\Phi^{-1}(0.5)}\right)$, where F represents the cumulative density function of the number-basis lognormal distribution (of the islet population), and Φ represents the cumulative density function of the standard normal distribution, $N(0,1)$.

Supplementary Table 7 Model parameters. Values and descriptions of constants used in all simulations.

Symbol	Description	Value (unit)
$\alpha_{O_2,h}$	Partial pressure-dependent solubility coefficient of oxygen in hydrogel	1.24×10^{-3} (mol m^{-3} $mmHg^{-1}$)
$\alpha_{O_2,c}$	Partial pressure-dependent solubility coefficient of oxygen in cell clusters	1.02×10^{-3} (mol m^{-3} $mmHg^{-1}$)
$\alpha_{O_2,t}$	Partial pressure-dependent solubility coefficient of oxygen in the nylon suture thread	1.90×10^{-3} (mol m^{-3} $mmHg^{-1}$)
$D_{O_2,h}$	Diffusion coefficient of oxygen in hydrogel	2.70×10^{-9} (m^2 s^{-1})
$D_{O_2,t}$	Diffusion coefficient of oxygen in nylon suture thread	5.00×10^{-13} (m^2 s^{-1})
$D_{O_2,c}$	Diffusion coefficient of oxygen in cell clusters	2.00×10^{-9} (m^2 s^{-1})
V_{max}	Maximum OCR (per unit volume) in cell clusters	See Supplementary Table 5
K_V	Half-maximal coefficient of oxygen consumption in cell clusters	0.80 (mmHg)
K_S	Half-maximal coefficient, oxygen-dependent insulin secretion potential	2.00 (mmHg)
n_S	Hill coefficient, oxygen-dependent insulin secretion potential	3 (unitless)
p_N	Threshold pO_2 required for cell survival (below which oxygen consumption and insulin secretion are ceased)	0.08 (mmHg)
p_{ext}	Boundary pO_2†	24–60 (mmHg)

†A default value of 40 mmHg was used (see discussion in the Supplementary Materials and Methods, Model implementation).

Supplementary Figures

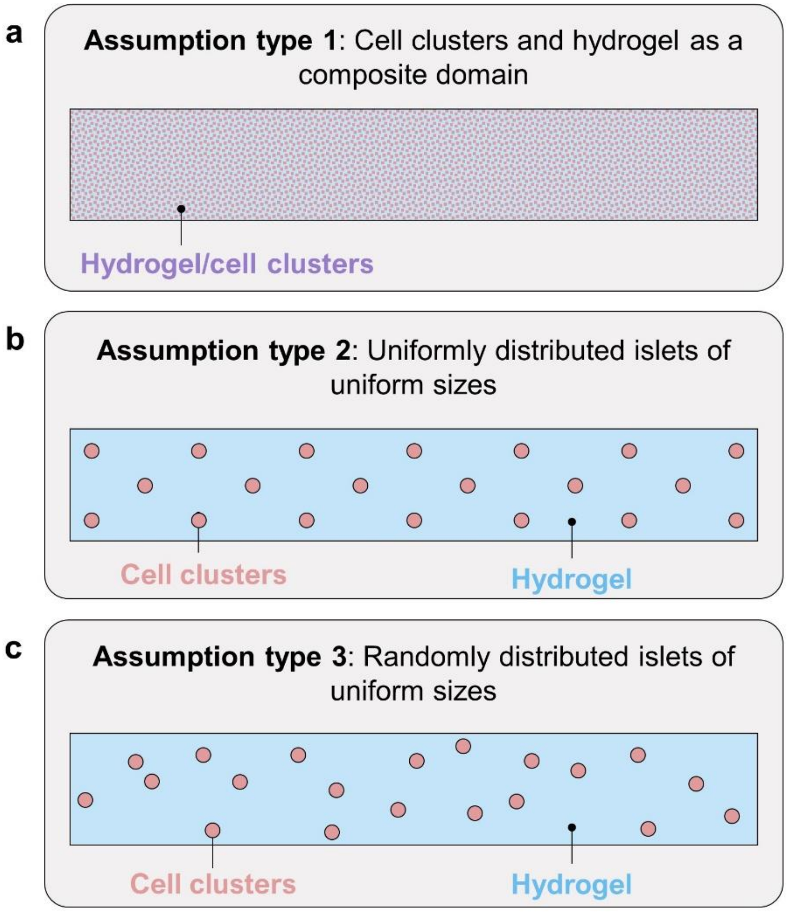

Supplementary Fig. 1 Geometry assumptions in BAP device models. a–c Models to simulate mass transport in islet encapsulation systems typically make one of three assumptions. The hydrogel and islets are considered as part of a composite domain with an effective OCR relative to the volume fraction of islets dispersed in the hydrogel (note, this is similar to assuming that constituent islet cells are uniformly distributed within the matrix) (**a**). Islets, of uniform diameter, are distributed in a uniform array in the hydrogel matrix (**b**). Islets, of uniform diameter, are distributed randomly in the hydrogel matrix (**c**). Models with these assumptions are likely to lead to more optimistic projections because they either do not account for the cells as clusters (Assumption Type 1), or do not consider the full range of islet sizes (Assumption Types 2 and 3).

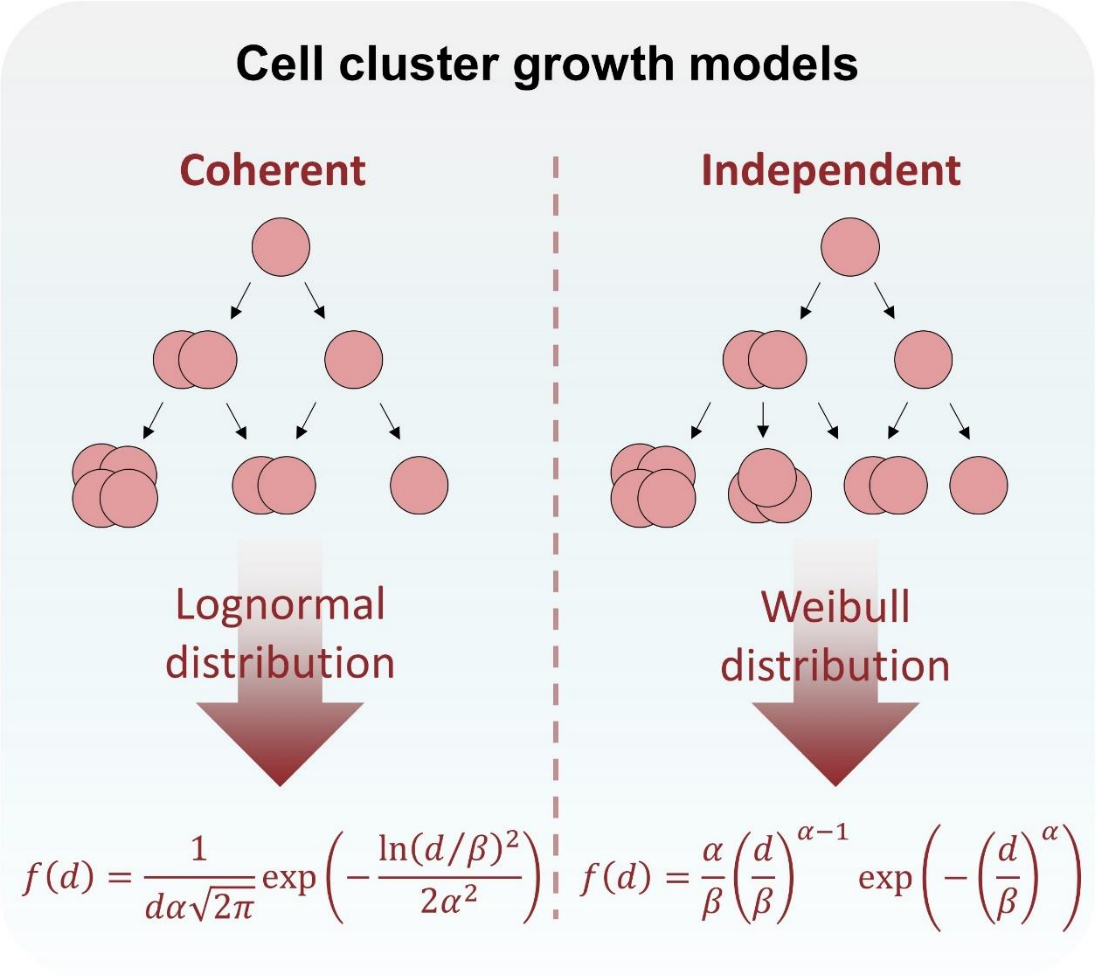

Supplementary Fig. 2 Cell proliferation dynamics in primary islets result in size distributions described by lognormal or Weibull functions. Cell proliferation via coherent dynamics (all cells replicate together or not at each replication time) results in cell clusters whose size distribution is described by the lognormal function, whereas cell proliferation via independent dynamics (cells replicate or not, independent of each other, at each replication time) result in cell clusters whose size distribution is described by the Weibull function[38]. Each circle represents a cell, and each horizontal row represents a discrete replication time.

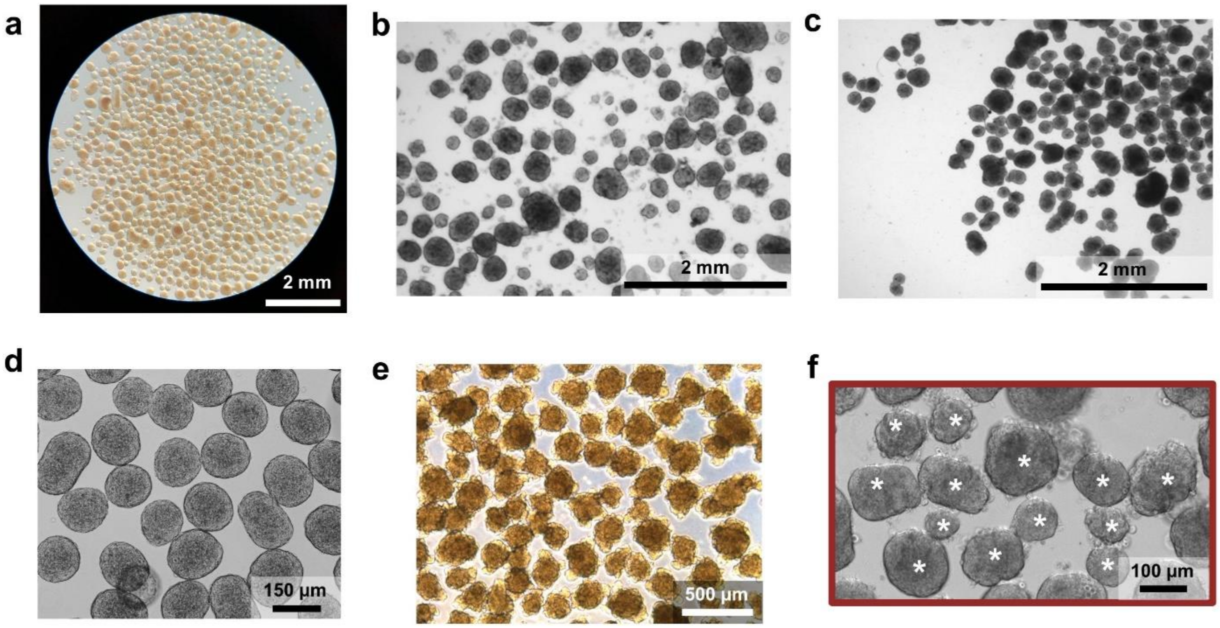

Supplementary Fig. 3 Representative islet images of islet isolations and SC-β preparations. a Stereo microscope image of isolated mouse islets (one of two shown). **b** Brightfield microscope image of isolated rat islets (one of 16 shown). **c** Brightfield microscope image of isolated juvenile porcine islets (one of 10 shown). **d** Brightfield microscope image of NN SC-βs (one of 20 shown). **e** Stereo microscope image of WU SC-βs (one of three shown). **f** Visual representation of cell cluster effective diameter measurement inclusion rules: (*i*) cell clusters partially cropped in the microscope image were not measured; (*ii*) cell clusters partially fused together were considered as separate entities (one of 16 shown). Each asterisk indicates a cell cluster which passed these criteria and were included in diameter measurement.

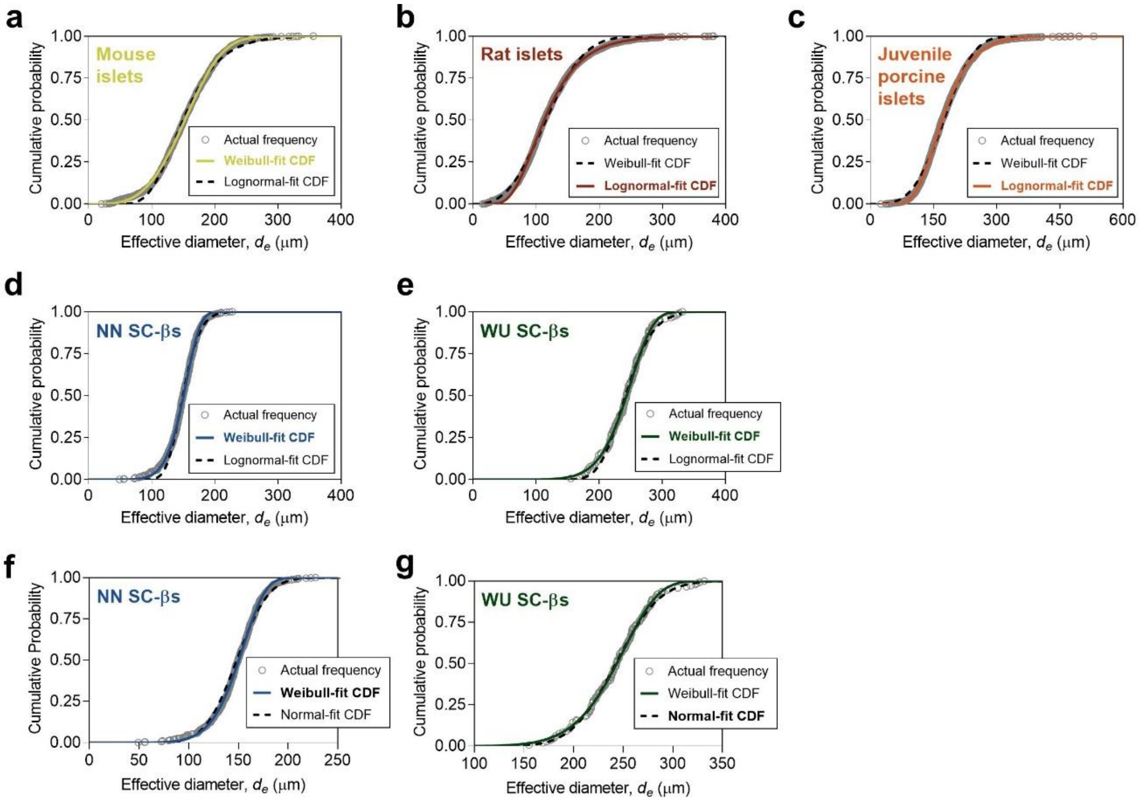

Supplementary Fig. 4 Size distribution curve fitting. a–e Empirical (actual) cumulative frequency of the effective diameters (d_e) of mouse islets (**a**), rat islets (**b**), juvenile porcine islets (**c**), NN SC-βs (**d**), WU SC-βs (**e**) (grey circles) versus the cumulative density function of the best fit lognormal and Weibull distributions (solid and dashed lines). The distribution type (i.e., Weibull versus lognormal) with the best fit is emphasized in bold in the figure legends. **f, g** Additional curve-fitting of NN SC-βs (**f**) and WU SC-βs (**g**) to normal distributions. The Weibull distribution was a more robust fit to the effective diameters of NN SC-βs than the normal distribution. The normal distribution was a more robust fit to those of WU SC-βs; however, the Weibull distribution was selected because of its still quite robust fit and nonexistence of positive probabilities for negative diameters. Source data are provided as a Source Data file.

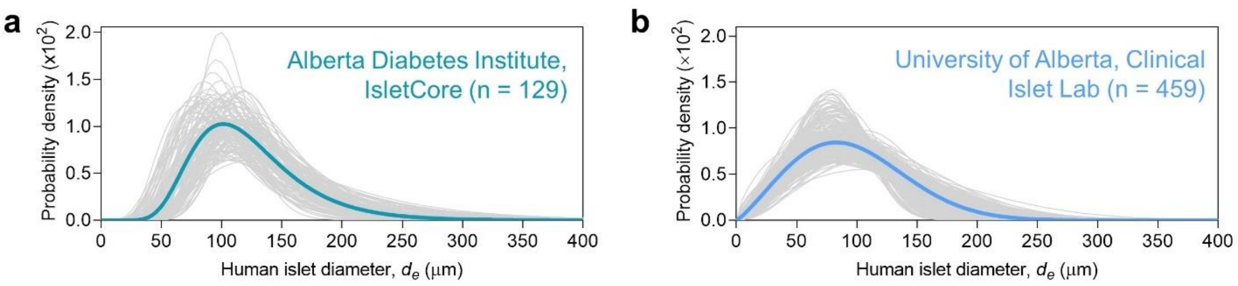

Supplementary Fig. 5 Human islet size distributions vary between isolation centers. a, b Probability densities of human islet effective diameters (d_e) collected at the IsletCore of the Alberta Diabetes Institute (**a**; n = 129) and the Clinical Islet Lab of the University of Alberta (**b**; n = 250 of 459 shown). Distributions from individual isolations are shown in grey and the distribution of the aggregated data is shown in color. Distributions of isolations from the Clinical Islet Lab are left-skewed in comparison with those from the IsletCore, showing that size distribution variance is in part a function of the isolation protocol, rather than exclusively the endogenous size distribution of islets within the native pancreas. Source data are provided as a Source Data file.

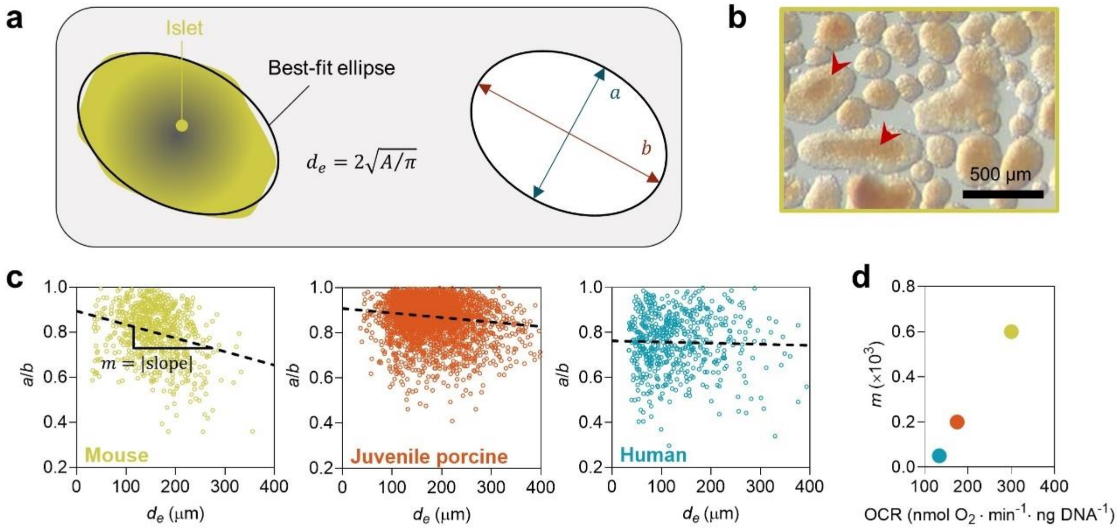

Supplementary Fig. 6 Islet ellipticity. a Definitions of the effective diameter d_e, minor diameter a, and major diameter b of the best-fit ellipse to an isolated islet. **b** Stereo microscope image of a representative mouse islet isolation, showing example large islets (red arrows) with high ellipticity (the representative image is a selected crop from Supplementary Fig. 3a). **c** Effective diameter d_e versus ellipticity, defined here as the ratio between the minor and major axes, a/b. Black dashed line shows best fit linear function with the slope magnitude, m. **d** Islet source reported oxygen consumption rates (OCR) versus slope magnitude in **c** (m); size-dependency of ellipticity is correlated to reported OCR, suggesting morphological trends toward higher ellipticity may be an adaptation to mitigate hypoxia. Source data are provided as a Source Data file.

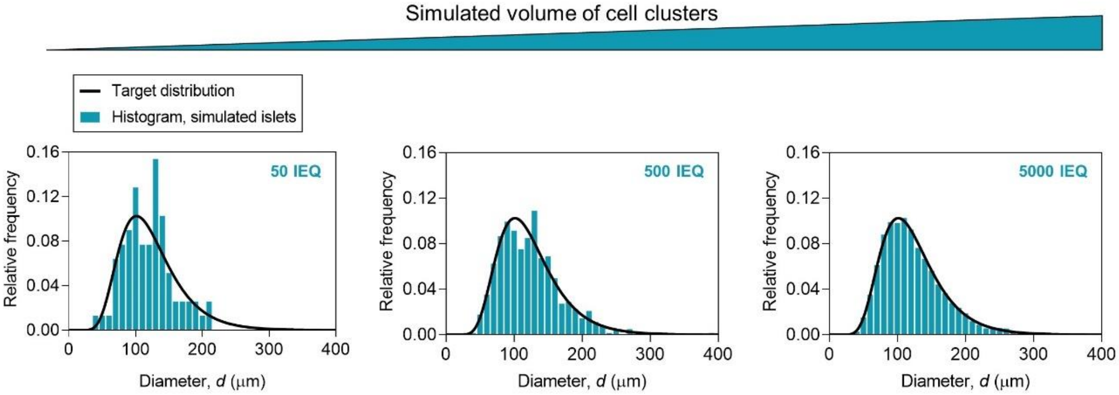

Supplementary Fig. 7 Simulated islet diameters converge to target distribution. Histogram of simulated islet diameters (blue bins) plotted with the probability density function of the target distribution (solid line) after 50 IEQ, 500 IEQ, and 5,000 IEQ of simulated islets (left to right), showing a convergence of the simulated diameters to the target distribution. Source data are provided as a Source Data file.

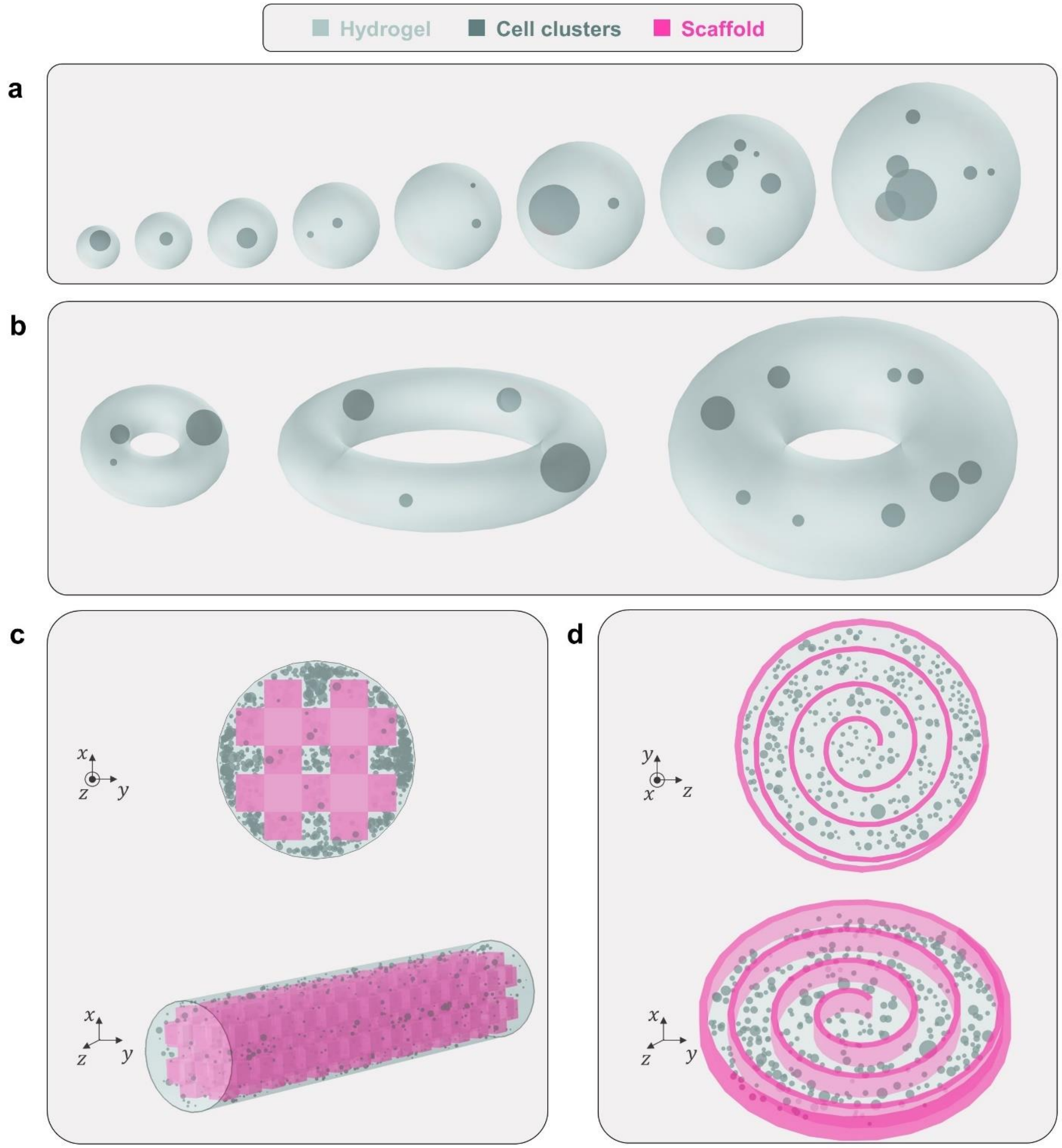

Supplementary Fig. 8 Example alternative geometries. a–d Device geometries other than those considered in the main text may be analyzed by SHARP, including simple parameterizable shapes such as spherical microcapsules (**a**), toroidal particles (**b**) or complex devices (**c** and **d**) with internal or external scaffolds.

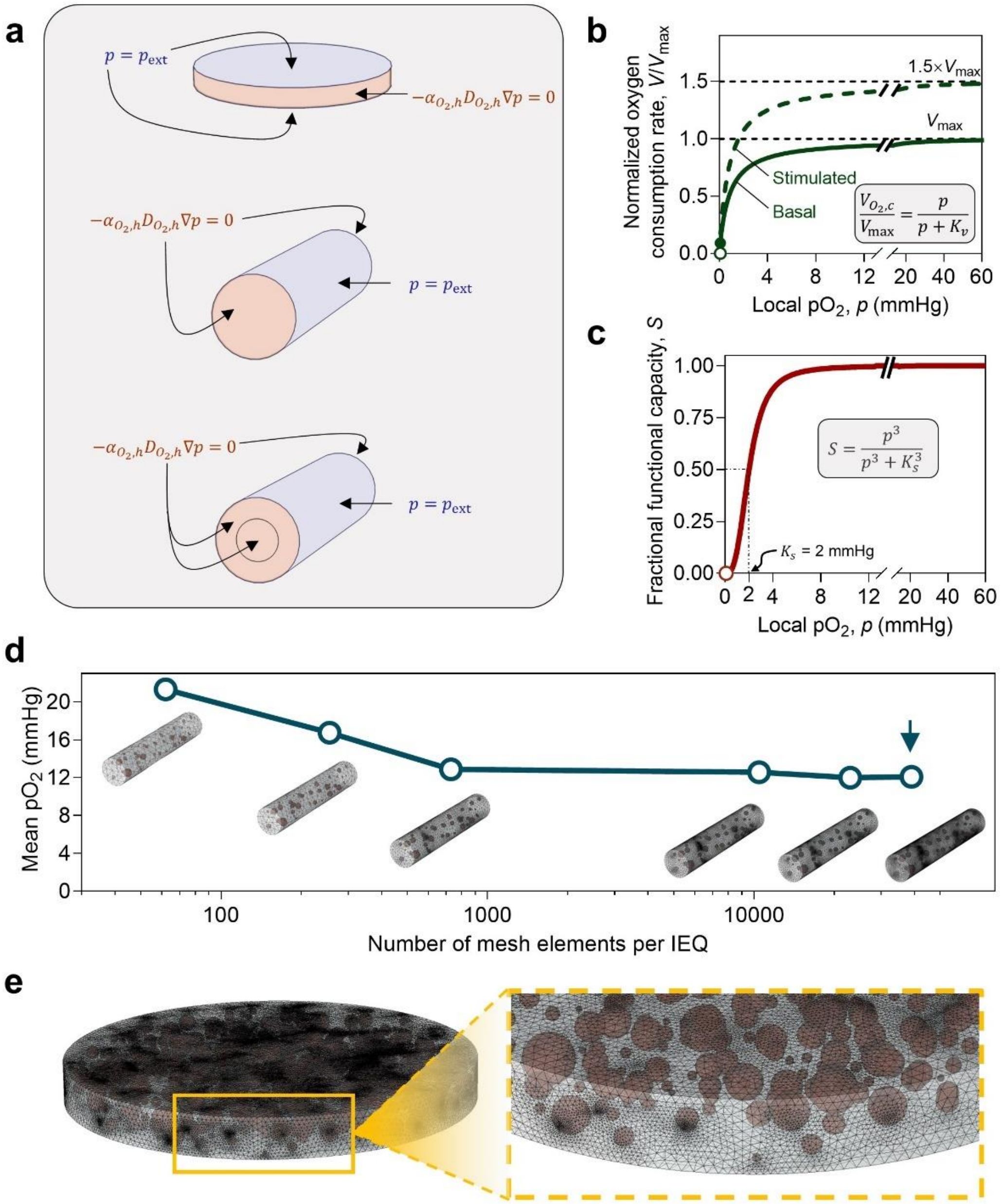

Supplementary Fig. 9 Model physics and finite element meshing. a Boundary conditions for (top to bottom) planar slab, cylindrical, and hollow cylindrical geometries. **b, c** Mathematical representations of oxygen consumption and loss of insulin secretion in islets/SC-βs. Normalized rate of oxygen consumption ($V_{O_2,c}/V_{max}$) in the cell clusters (**b**) under basal (low glucose) and stimulated (high glucose) conditions as a function of the local pO_2, p; the discontinuity at $p = p_N$ = 0.08 mmHg represents the arrestation of oxygen consumption due to necrosis. Local fractional second-phase insulin secretion capacity (**c**), S, as a function of p; at 2 mmHg, S is at half of the level under fully oxygenated conditions. Open circle represents discontinuity in tissue regions at p < 0.08 mmHg, which are assumed to be necrosed and therefore fully nonfunctional (S = 0). In the main text, the fractional loss of insulin secretion capacity, Ψ, is reported, which is defined as mean value of S in the cell cluster (or population of cell clusters) subtracted from 1. **d** Mesh

convergence analysis: number of mesh elements (normalized to the islet volume, in IEQ) versus mean pO_2 of the islet population in a cylindrical construct (50 IEQ, 1 mm diameter). The arrow indicates the mesh fineness implemented in all studies. **e** Representative image of the free-tetrahedral, nonuniform finite element mesh over which oxygen distributions are calculated. Source data are provided as a Source Data file.

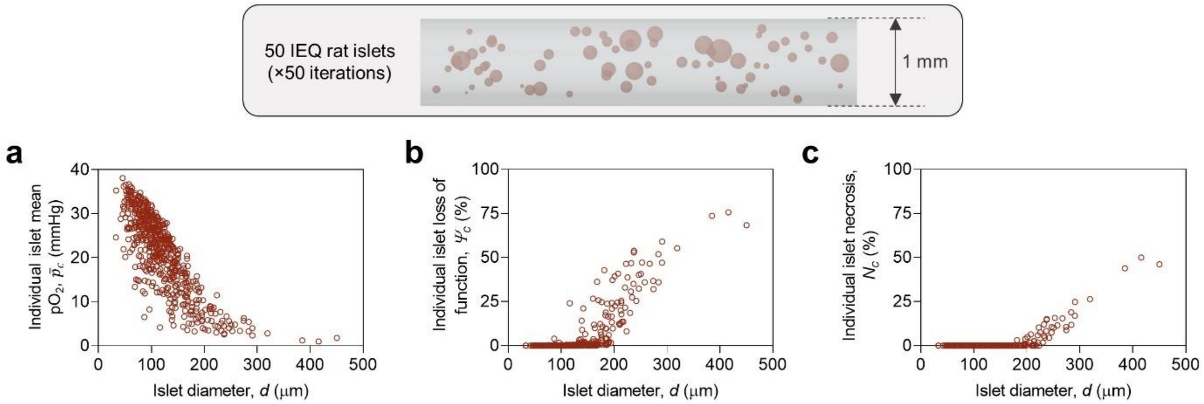

Supplementary Fig. 10 Effect of islet size on oxygenation outcomes in a bioartificial pancreas device. a–c Mean pO_2 (**a**), loss of function (**b**), and necrosis percentage (**c**) versus islet diameter in a hypothetical cylindrical construct (described in the schematic above). Aggregated data is displayed in Fig. 2e–h (n = 571 simulated rat islets). A clear negative relationship is observed between islet size and oxygenation, and a positive correlation is observed between islet size and loss of function and necrosis. Source data are provided as a Source Data file.

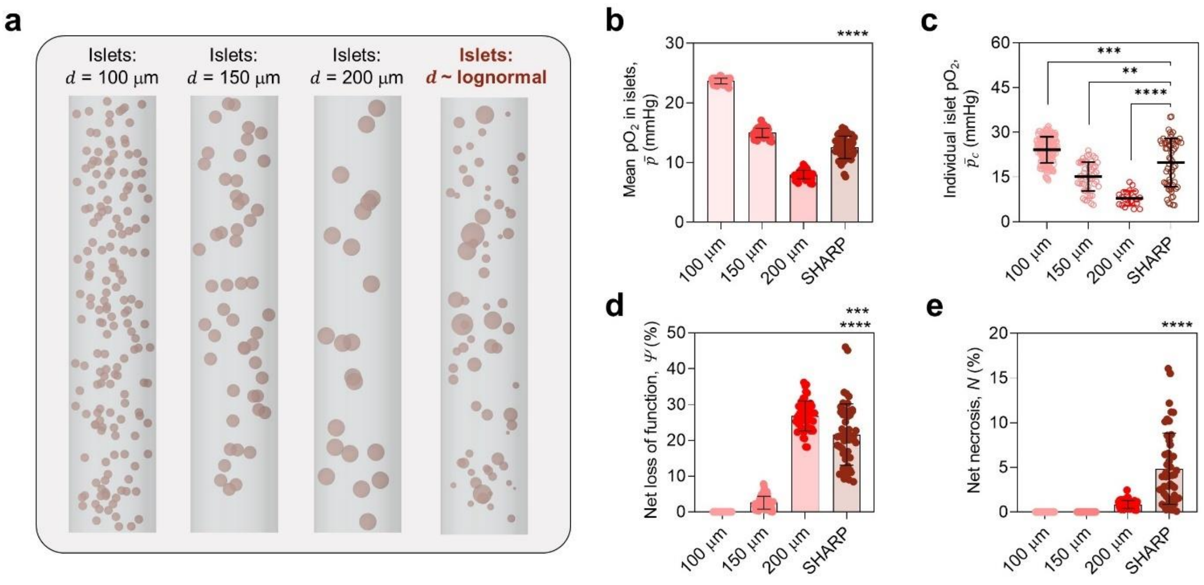

Supplementary Fig. 11 Biases of uniform islet size assumptions. a Schematic illustrating study design: 50 IEQ (2.25%, v/v) in 1 mm diameter cylinders, comparing the effect of implementing all islets as 100, 150, or 200 μm in diameter, or randomly selected from their empirical lognormal distribution via SHARP. The simulation was reiterated 50 times, each time rerandomizing the islet positions (and for SHARP, the islet size selections). **b** Mean pO_2 of the islet population versus islet size assumption. *****p* = 5.2e-42 (100 μm group [n = 50 iterations] versus SHARP [n = 50 iterations]), *****p* = 2.3e-11 (150 μm group [50 iterations] versus SHARP), *****p* = 2.8e-23 (200 μm group [n = 50 iterations] versus SHARP). **c** Mean pO_2 in individual islets versus islet size assumption, selected from one iteration. The open circles indicate values for individual islets, and the black lines represent mean ± SD; ****p* = 0.0010 (100 μm group [n = 169 islets] versus SHARP [n = 62 islets]); ***p* = 0.0015 (150 μm group [n = 50] versus SHARP); *****p* = 4.8e-16 (200 μm group [n = 22] versus SHARP). **d** Fractional loss of insulin secretion, Ψ, expected according to each islet size assumption, showing that assuming islets to be ≤ 150 μm under-predicts this metric.; ****p* = 0.0008 (200 μm group versus SHARP); *****p* = 1.0e-20 (150 μm group versus SHARP); n = 50 iterations. All values for the 100 μm group were 0 and thus could not be evaluated statistically. **e** Necrotic islet volume, N, versus islet size assumption, showing that even the 200 μm group assumption underestimates the degree of necrosis, which occurs in larger islets. *****p* = 4.6e-11 (150 μm group versus SHARP), *****p* = 9.5e-9 (200 μm group versus SHARP); n = 50 iterations. All values for the 100 μm group were 0 and thus could not be evaluated statistically. Statistical analysis in **b**–**e** was performed using Brown-Forsythe and Welch ANOVA with two-sided Games-Howell post hoc *p*-value adjustment for multiple comparisons. Source data are provided as a Source Data file.

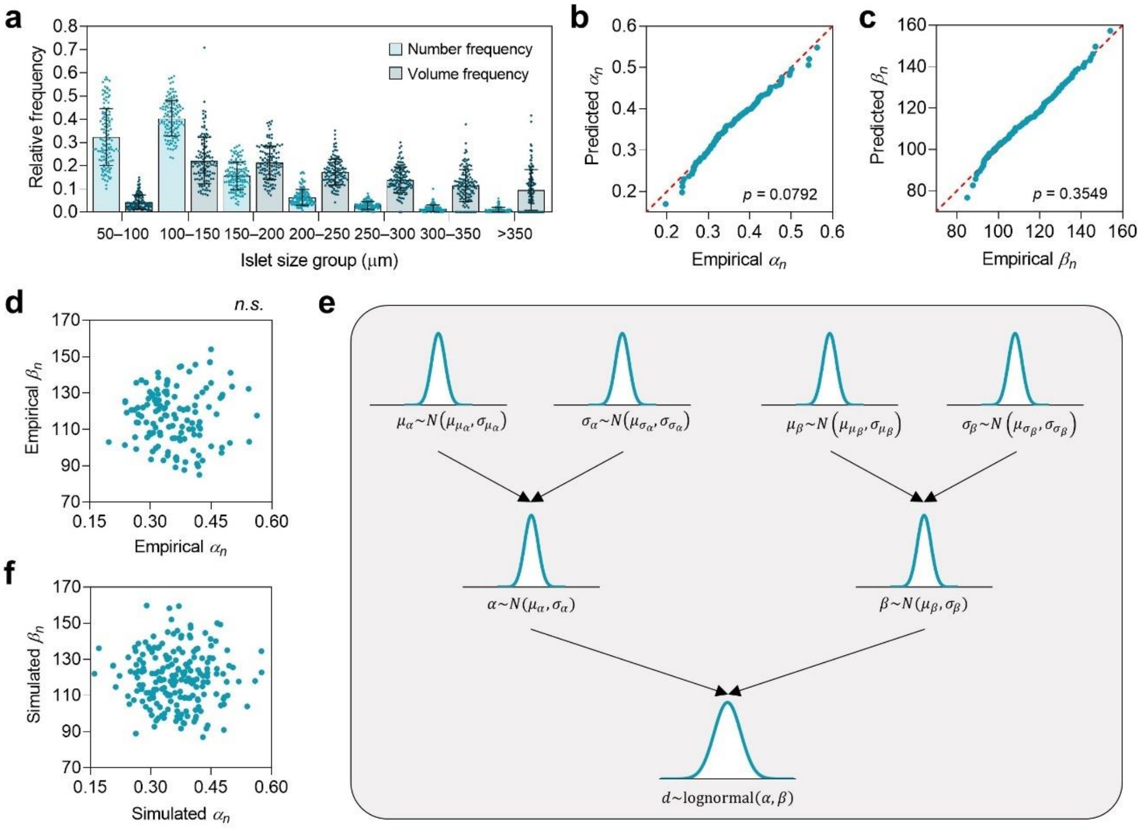

g Hyperparameter values.

First level	Second level	Third level
$\alpha \sim N(\mu_\alpha, \sigma_\alpha)$	$\mu_\alpha \sim N(\mu_{\mu_\alpha}, \sigma_{\mu_\alpha})$	$\mu_{\mu_\alpha} = 3.590 \times 10^{-1}$
		$\sigma_{\mu_\alpha} = 6.231 \times 10^{-3}$
	$\sigma_\alpha \sim N(\mu_{\sigma_\alpha}, \sigma_{\sigma_\alpha})$	$\mu_{\sigma_\alpha} = 7.077 \times 10^{-2}$
		$\sigma_{\sigma_\alpha} = 4.406 \times 10^{-3}$
$\beta \sim N(\mu_\beta, \sigma_\beta)$	$\mu_\beta \sim N\left(\mu_{\mu_\beta}, \sigma_{\mu_\beta}\right)$	$\mu_{\mu_\beta} = 1.170 \times 10^{2}$
		$\sigma_{\mu_\beta} = 1.327$
	$\sigma_\beta \sim N\left(\mu_{\sigma_\beta}, \sigma_{\sigma_\beta}\right)$	$\mu_{\sigma_\beta} = 1.507 \times 10^{1}$
		$\sigma_{\sigma_\beta} = 9.380 \times 10^{-1}$

Supplementary Fig. 12 Human islet size distribution characterizations. a Histogram of the relative frequency (on number and volume bases) of human islet diameters (n = 129 isolations). Bars and error bars represent mean ± SD, respectively. **b, c** Quantile-Quantile (QQ) plots of best-fit number-distribution shape parameters α_n (**b**) and scale parameters β_n (**c**) versus those as predicted by normal distributions (n = 129). Each blue circle represents a parameter value from one distribution and the line of identity (dashed, red) represents a theoretical normal distribution; *p*-values from a Shapiro-Wilk normality test (where p > 0.05 indicates normality) are shown. **d** Scatter plot of empirically determined α_n versus β_n for all isolations (n = 129), showing no correlation between them. *n.s.* (p = 0.0832; two-sided Pearson's correlation, r = 0.02). **e** Visual illustration of human islet size distribution simulation by the hierarchical probabilistic Monte Carlo model: each parameter was simulated independently (first level), assumed to be described by normal

distributions (second level), each with some error associated with the mean, μ, and standard deviation, σ, of the best-fit normal distribution (third level). **f** Scatter plot of α_n versus β_n (n = 200) for simulated distributions, which resembled the empirically determined values. **g** Tabulated hyperparameter values. To summarize, the hierarchical probabilistic Monte Carlo model was developed as follows. (*i*) Lognormal distributions were fit to all 129 human islet isolations individually. (*ii*) Parameter sets α_n and β_n were subjected to normality tests (**b** and **c**), through which they were found to be appropriately described by the normal distribution, and then a correlation test (**d**) which found them to be uncorrelated. (*iii*) Normal distributions were fit to parameter sets α_n and β_n via the maximum likelihood approach, which determined the absolute values of the third level hyperparameters. Source data are provided as a Source Data file.

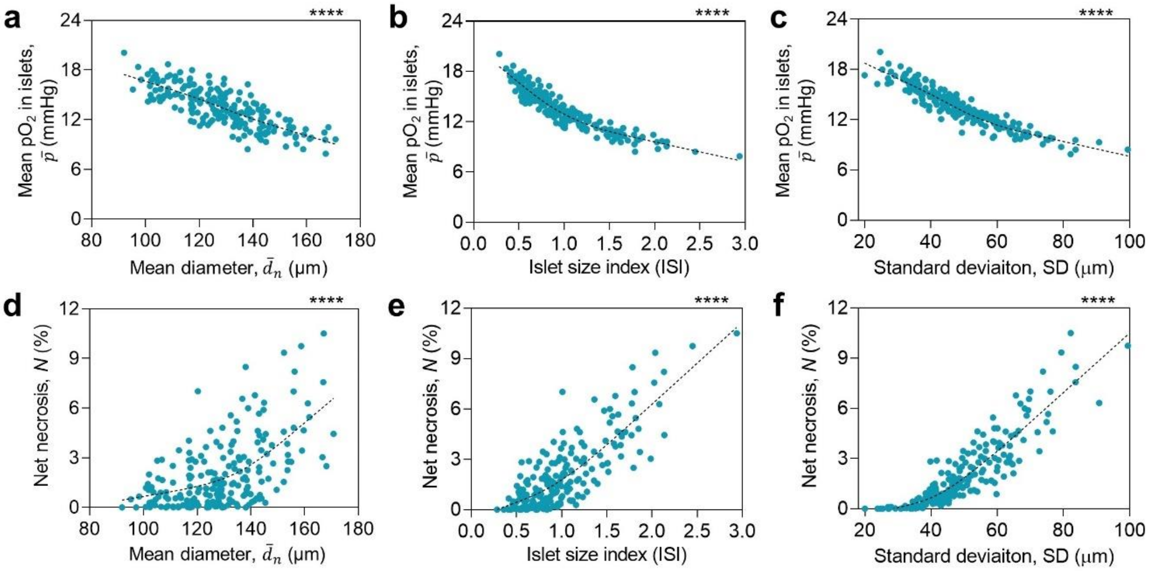

Supplementary Fig. 13 Encapsulated human islet outcomes are predictably dependent on size distribution properties. a–c Mean pO_2 of the islet population versus mean number-basis diameter (**a**), islet size index (**b**), and standard deviation (**c**) of simulated human islet distributions in hypothetical construct described in Fig. 3e. **d–f** Net necrosis percentage versus mean number-basis diameter (**d**), islet size index (**e**), and standard deviation (**f**). **a**: ****p = 2.7e-42, **b**: ****p = 7.2e-75, **c**: ****p = 3.2e-84, **d**: ****p = 7.0e-18, **e**: ****p = 4.9e-49, **f**: ****p = 3.8e-68; two-sided Pearson's correlation (n = 200 simulated devices). The dashed line represents the best-fit spline curve. Source data are provided as a Source Data file.

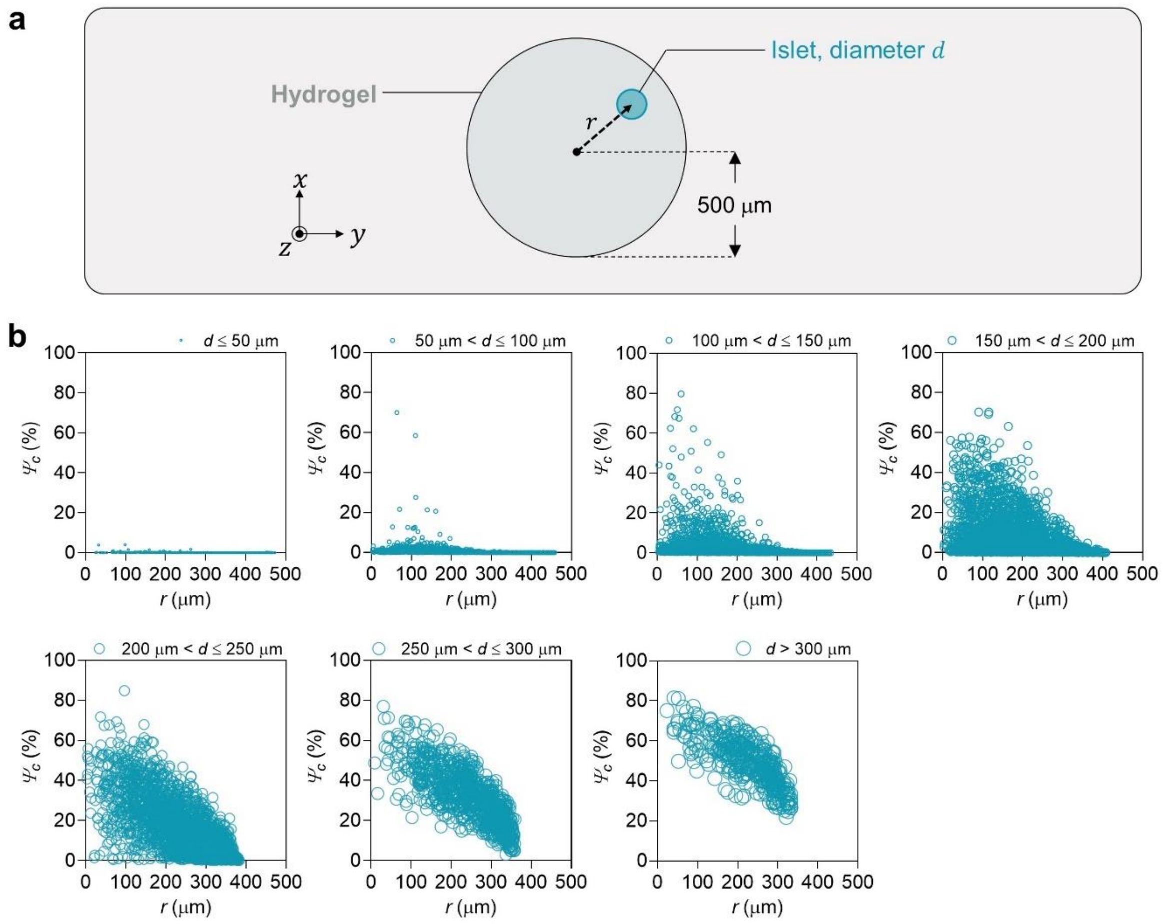

Supplementary Fig. 14 Position dependency of islet outcomes. a Schematic showing the measurement of the radial position, r, defined as the distance from the center line of the cylinder device to the center of the islet. **b** Scatter plots of r versus fractional loss of function of individual islets (Ψ_c) for islets within variable size ranges (indicated by the top caption). Centrally located islets showed higher impairment than more peripherally located ones. Source data are provided as a Source Data file.

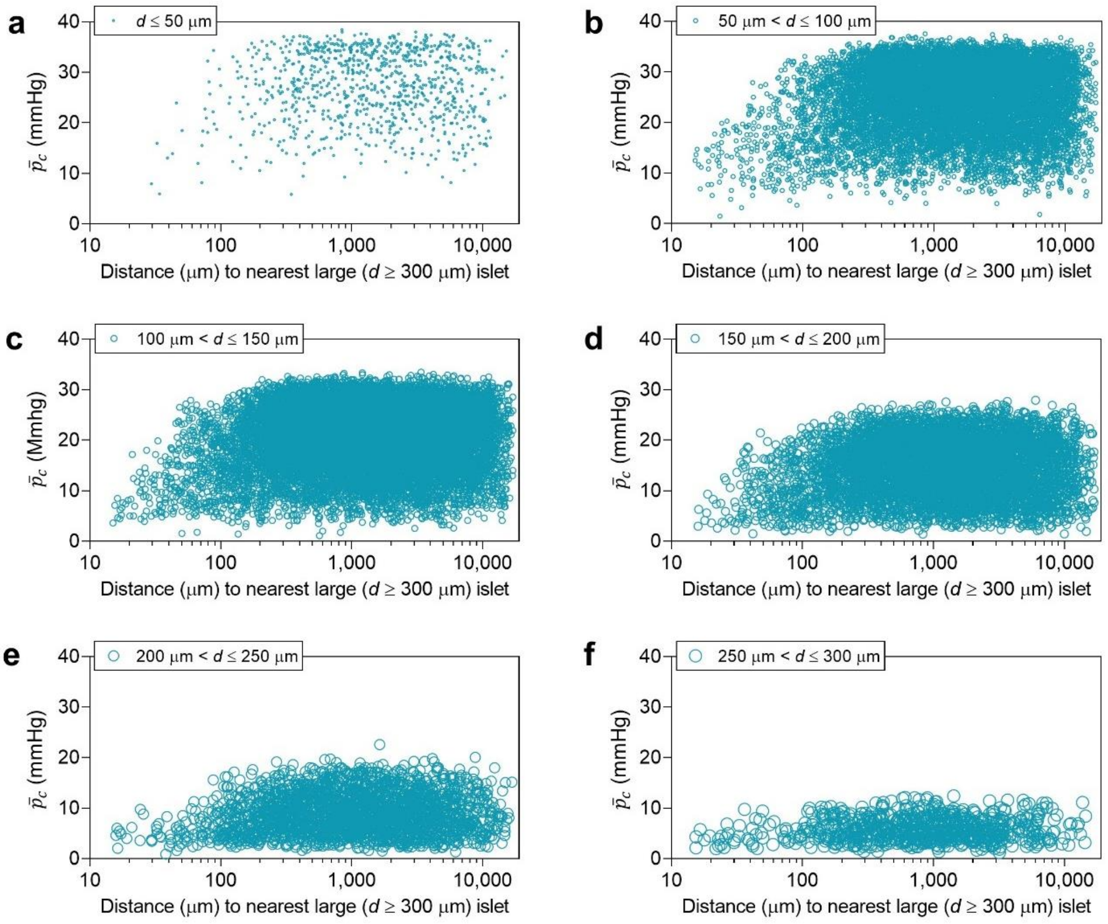

Supplementary Fig. 15 Large islets diminish the oxygenation of nearby smaller ones. a–f Mean islet pO_2 within a reference islet ($\bar{p}_c$) versus distance to nearest islet $d \geq 300$ μm for islets of different size ranges (indicated by the top caption). Reference islets in all size buckets show reduced oxygen levels when within ~100 μm of an islet $d \geq 300$ μm, showing that large islets also impact performance by constricting oxygen flow to smaller ones. Source data are provided as a Source Data file.

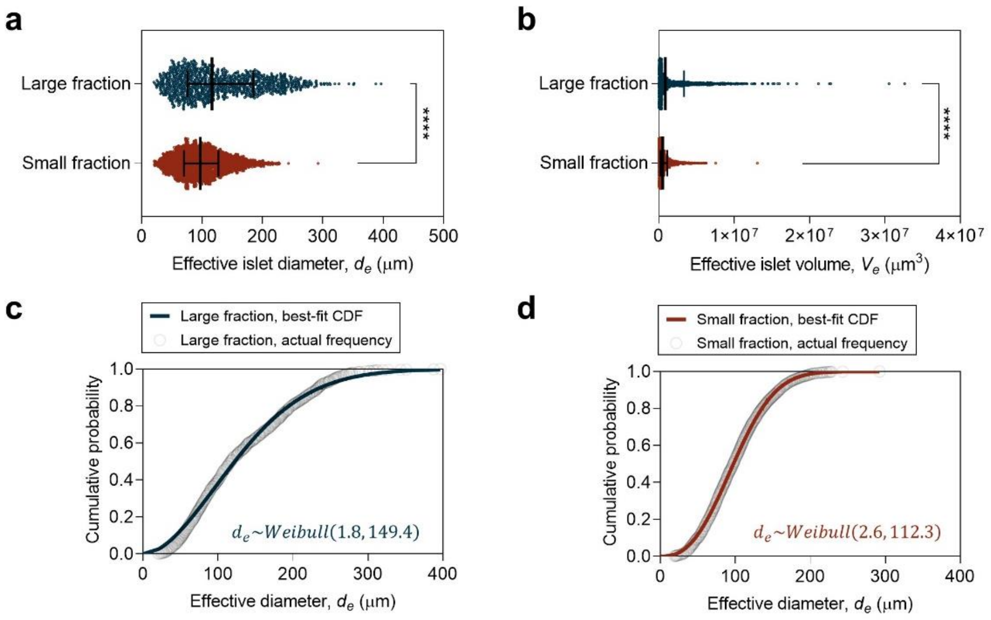

e Rat islet fraction size distribution properties post-partitioning.

Parameter	Large fraction	Small fraction
Islet size index (ISI)	1.3	0.5
Number distribution shape parameter, α_n	1.8	2.6
Volume distribution shape parameter, α_v	4.2	5.3
Number distribution scale parameter, β_n	149.4	112.3
Volume distribution scale parameter, β_v	243.0	139.6
Number-mean diameter, $\bar{d}_n$ (μm)	132.8	99.7
Volume-mean diameter, $\bar{d}_v$ (μm)	220.9	128.5
Number distribution standard deviation, SD_n	75.7	41.9
Volume distribution standard deviation, SD_v	59.1	28.1

Supplementary Fig. 16 Characterization of artificially manipulated rat islet fractions. a, b Effective diameters (d_e) (**a**) and effective volumes (V_e) (**b**) of isolated rat islets after partitioning into large (n = 1,171) and small (n = 3,120) fractions. Circles indicate actual values; black lines indicate median ± interquartile range. **a** and **b**: ****p = 2.2e-33; unpaired two-sided Mann-Whitney test. **c, d** Curve fitting fraction d_e distributions: number-basis actual cumulative frequency (open circles) versus best-fit Weibull cumulative density function (solid lines) for the large fraction islets (**c**) and small fraction islets (**d**), respectively. **e** Size distribution properties of each fraction. According to the analysis provided in Supplementary Table 6, all distribution properties favor the outcomes of the small fraction. Source data are provided as a Source Data file.

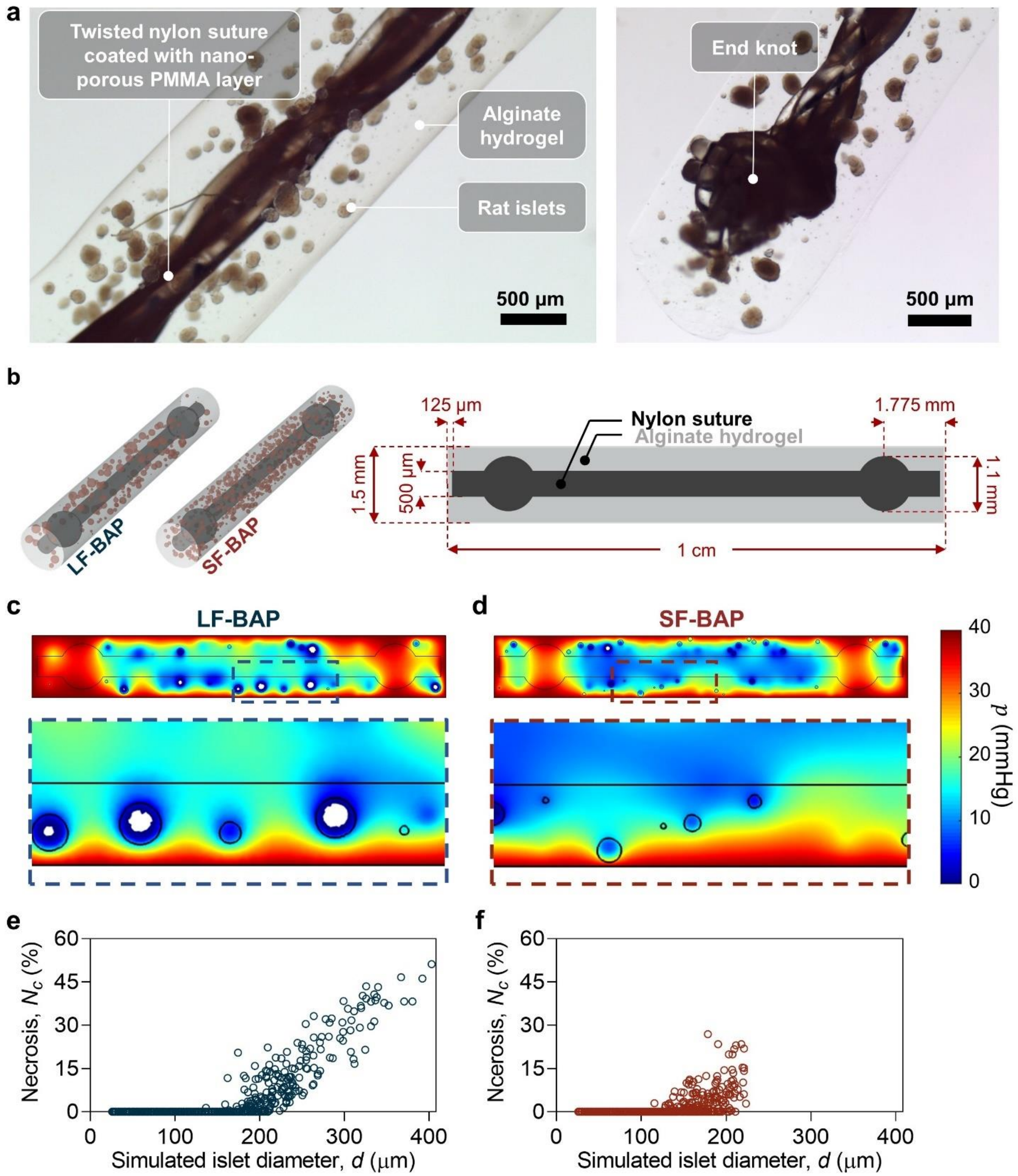

Supplementary Fig. 17 Rat islet-containing BAPs. a Representative stereo microscope images of the model cell encapsulation device used for testing the influence of islet size distributions on diabetes corrective capacity; the image on the right shows a representative image of the knot at the end of the twisted suture used to maintain torsion in the thread, integrated into the model as a sphere near the end of the thread. **b** Schematics (of the LF-BAP and SF-BAP, left and right, respectively) and dimensions used in

SHARP to simulate the model BAP. **c, d** Surface plots showing the spatial pO_2 (p) distribution in a center cut plane of one iteration of the LF-BAP (**c**) and SF-BAP (**d**) simulations. White space within simulated islets indicates regions of expected necrosis. **e, f** SHARP-predicted necrotic volume percentage, N_c, versus islet diameter, d, of individual islets in the LF-BAPs (n = 801) (**e**) and in the SF-BAPs (n = 2,576) (**f**); data shown were collected from 4 iterations of each device containing an islet volume of 300 IEQ (aggregated data shown in Fig. 4h). Source data are provided as a Source Data file.

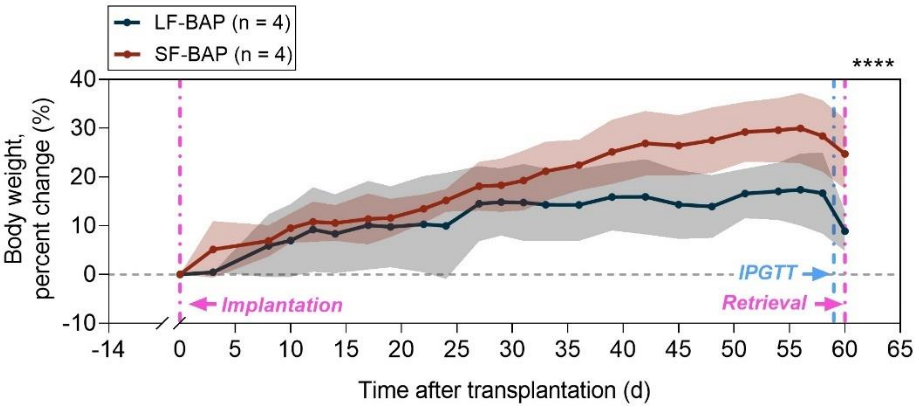

Supplementary Fig. 18 Body weight of mice during diabetes reversal test. Percent change in body weight in STZ-induced diabetic C57BL/6J mice after implantation of large fraction-containing devices (LF-BAP, n = 4) and small fraction-containing devices (SF-BAP, n = 4). The IPGTT, conducted at day 59, was preceded by an overnight fasting period, resulting in a drop in body weight for both groups, which did not fully recover by the time of retrieval on day 60. Lines show mean values and error bands represent ± SD. ****p = 1.4e-10; one-way ANCOVA. Source data are provided as a Source Data file.

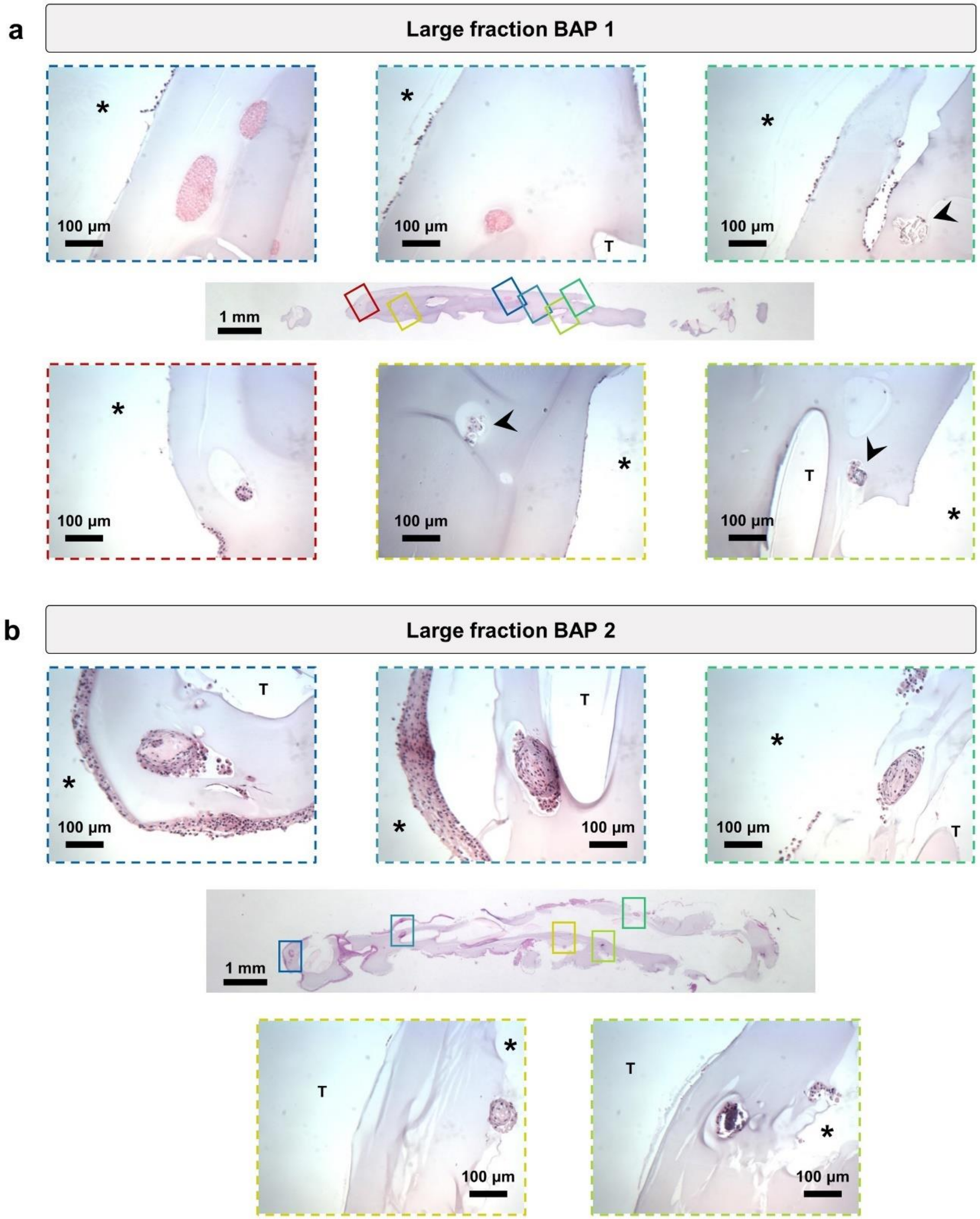

Supplementary Fig. 19 (Continued on the following page).

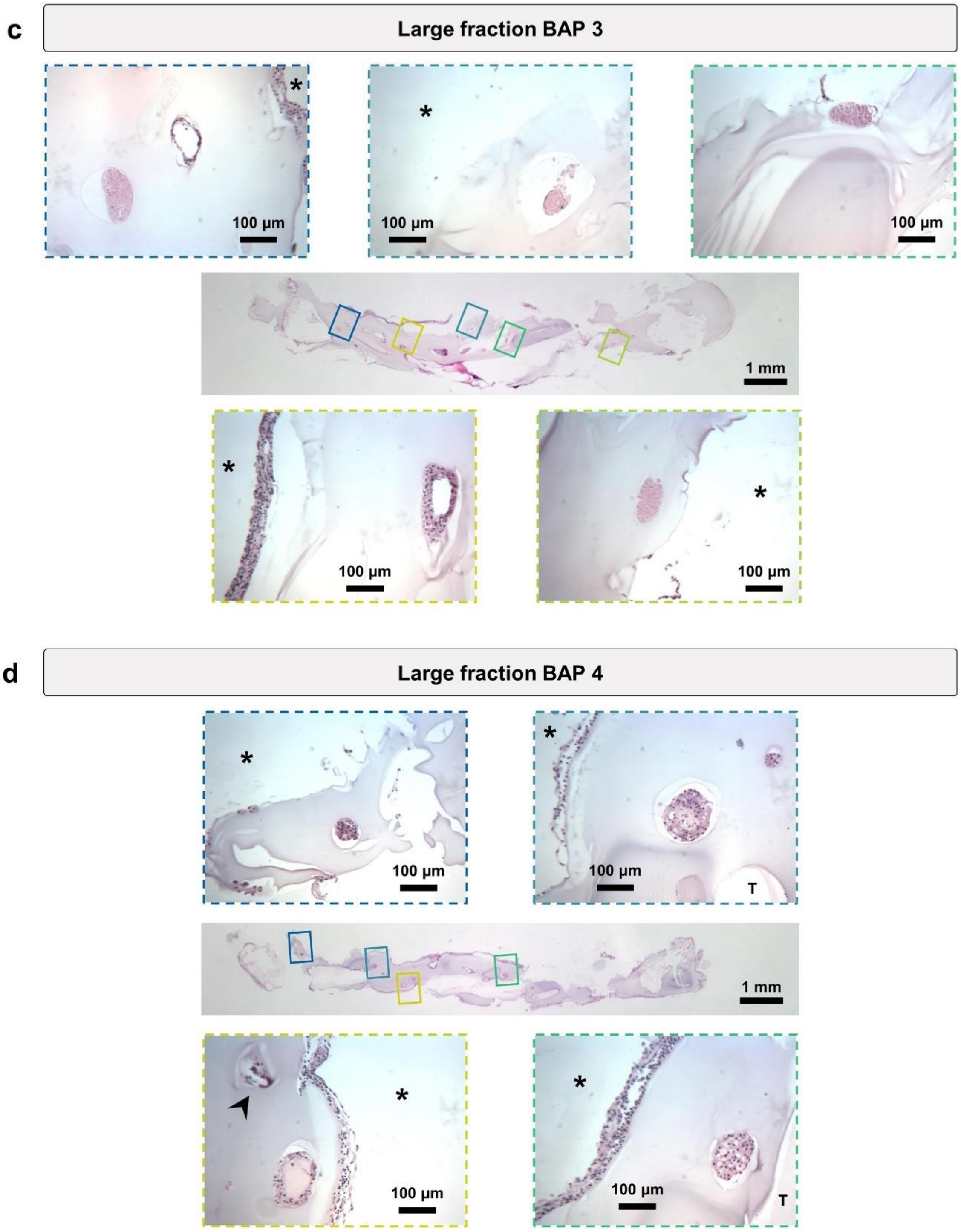

Supplementary Fig. 19 Histology, large fraction-containing encapsulation devices (LF-BAPs). a–d Microscope images of H&E-stained retrieved LF-BAPs. One full section is shown with several magnifications highlighting individual islets (**a**: 7 of 20, **b**: six of 14; **c**: five of 18; and **d** five of 25 shown). Several completely denucleated islets were observed, as well as many islets with viable peripheries but necrotic cores. Asterisks indicate the device side of the device-host boundary, T symbols represent the internal thread, and black arrows highlight regions which are possibly completely necrosed islets.

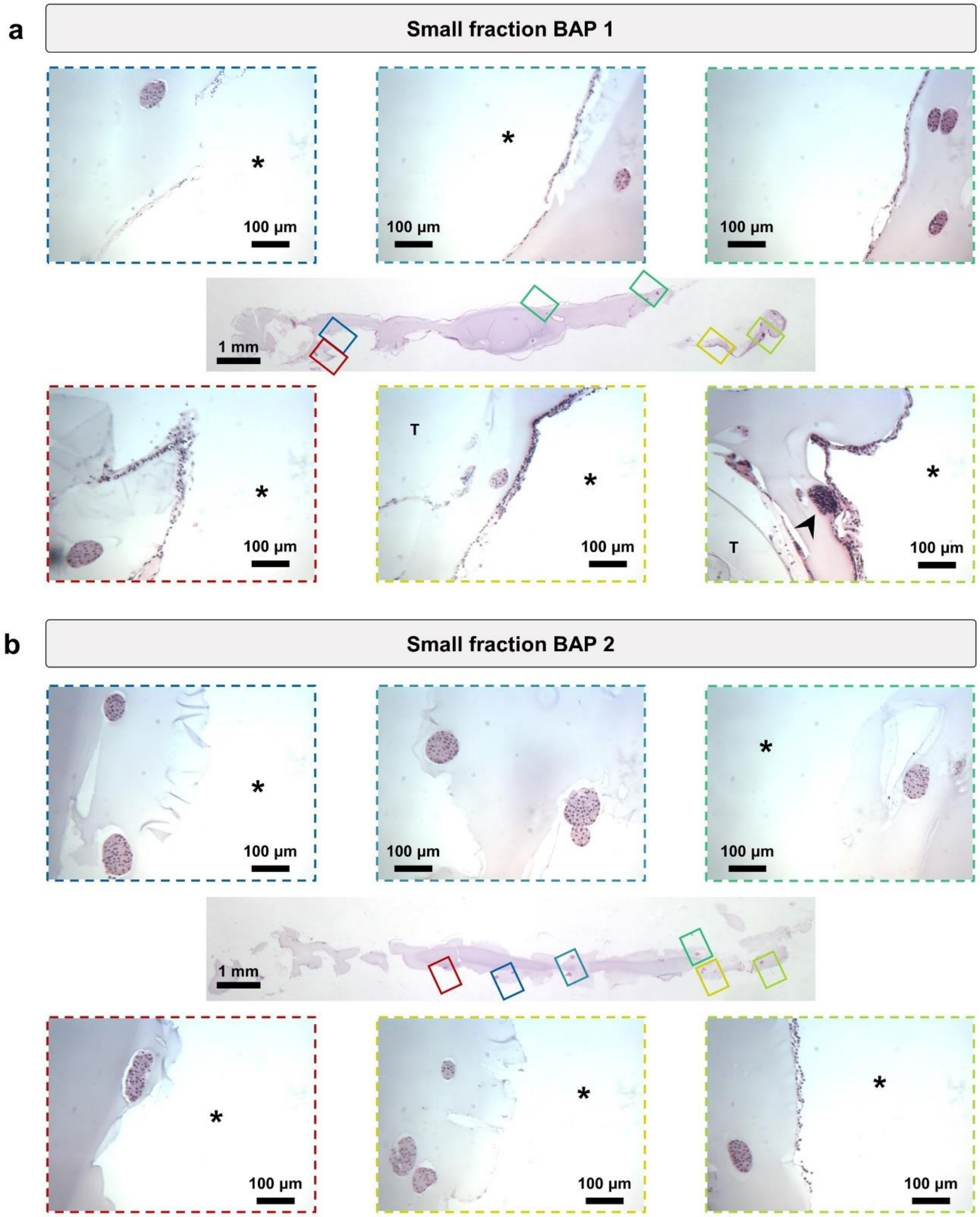

Supplementary Fig. 20 (Continued on the following page).

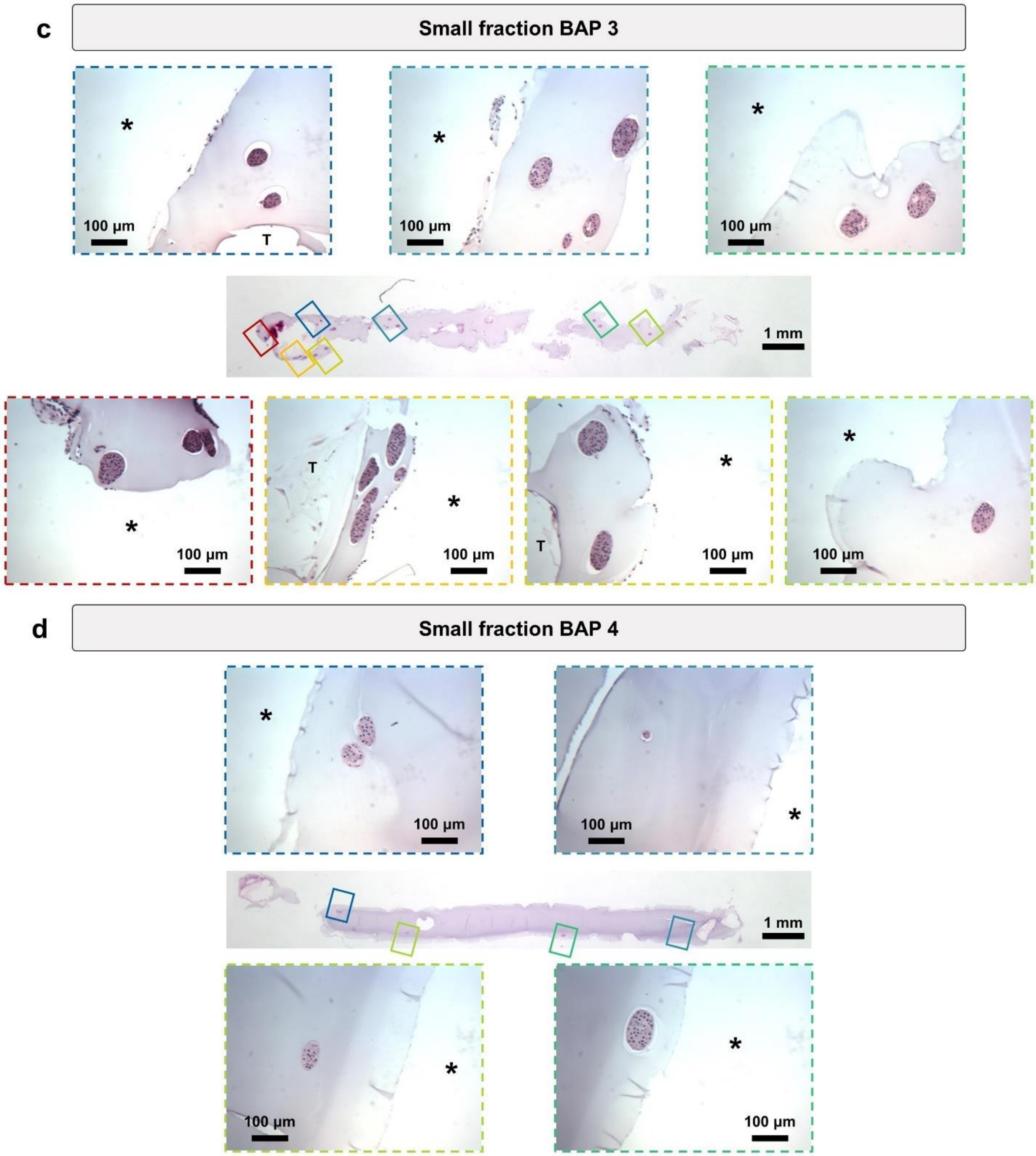

Supplementary Fig. 20 Histology, small fraction-containing encapsulation devices (SF-BAPs). a–d Representative microscope images of H&E-stained retrieved SF-BAPs. One full section is shown with several magnifications highlighting individual islets (**a**: 7 of 37, **b**: 7 of 42; **c**: 8 of 23; and **d** five of 15 shown). Most observed islets were completely viable, though some larger islets exhibited viable peripheries with karyorrhexis or necrosis in the core cells. Asterisks indicate the host side of the device-host boundary and T symbols represent the internal thread.

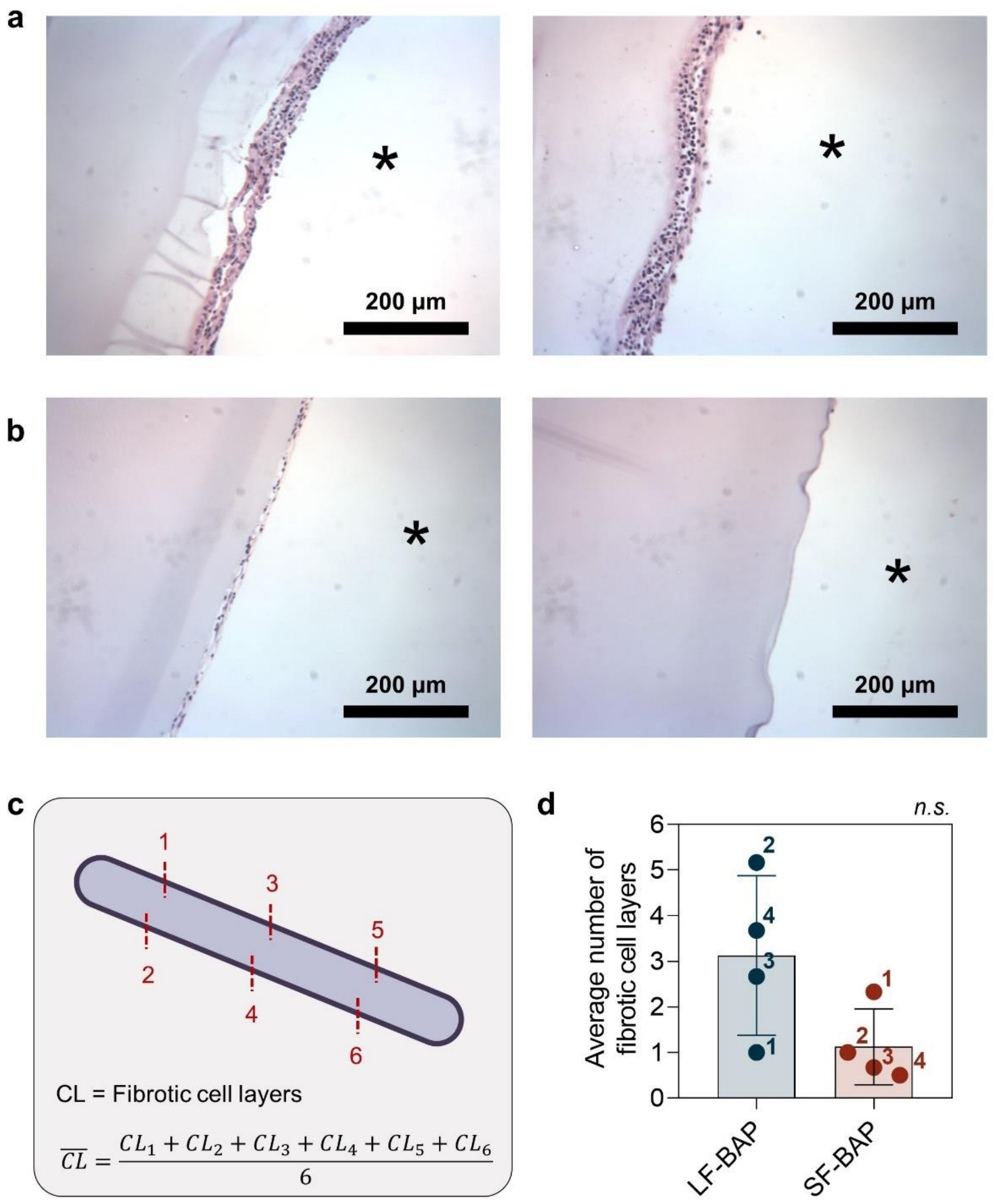

Supplementary Fig. 21 Fibrosis characterization. a, b Representative microscope images of H&E-stained retrieved LF-BAPs (**a**) (two of four shown) and SF-BAPs (**b**) (two of four shown), highlighting the variable fibrotic coverage. Asterisks indicate the host side of the device-host interface. **c** Fibrosis quantification rules: the number fibrotic cell layers at 6 sites (as indicated in the illustration) were counted and averaged. **d** Average fibrotic cell layers on the retrieved LF-BAPs and SF-BAPs (data shown represent mean ± SD for n = 4 devices from each group; each closed circle corresponds to one device indicated by the number offset to the right). Note, device numbers refer to histological images in Supplementary Figs. 19 and 20 (e.g., SF-BAP 1 and LF-BAP 1 have no relation). *n.s.* (p = 0.0844); unpaired two-sided student's *t*-test. Source data are provided as a Source Data file.

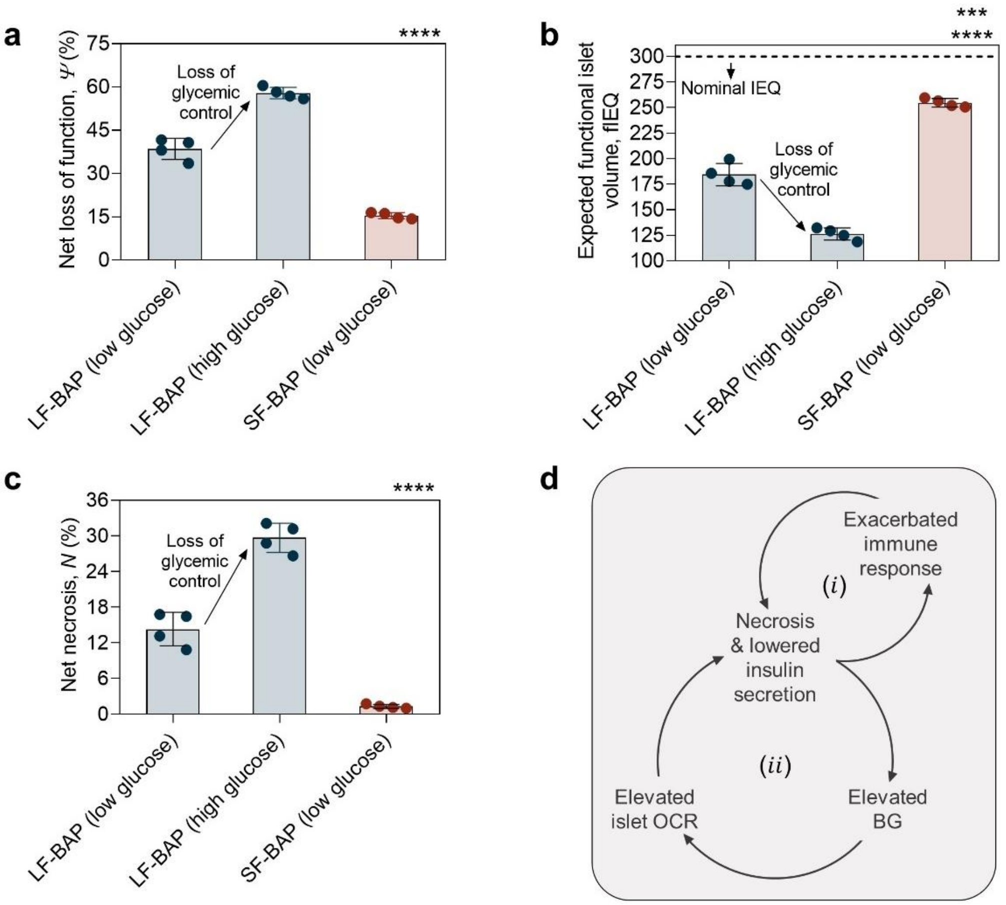

Supplementary Fig. 22 Theoretical basis for proposed feedback mechanism between hyperglycemia and graft attrition in mice. a–c SHARP-predicted net fractional loss of function (**a**), expected fIEQ (**b**), and necrosis (**c**) in the LF-BAP under low and high glucose conditions and the SF-BAP under low glucose conditions (n = 4). Islet OCR is higher under high glucose conditions, hence functional outcomes are expected to be worse. **d** Proposed feedback between mouse hyperglycemia and deteriorating graft function in the induced diabetic mouse model: (*i*) necrosis in islets exacerbates the immune stressors; (*ii*) necrosis and poor insulin secretion lead to elevated BG levels, which elevates the OCR of the encapsulated islets, resulting in higher levels of necrosis and even poorer insulin secretion capacity. Data are presented as mean ± SD. **a**: ****p = 4.6e-6 (LF-BAP, low glucose versus high glucose), ****p = 1.0e-6 (LF-BAP [low glucose] versus SF-BAP), ****p = 5.0e-9 (LF-BAP [high glucose] versus SF-BAP). **b** ***p = 0.0003 (LF-BAP, high glucose versus low glucose), ****p = 1.1e-6 (LF-BAP [low glucose] versus SF-BAP), ****p = 5.5e-9 (LF-BAP [high glucose] versus SF-BAP). **c**: ****p = 2.4e-6 (LF-BAP, high glucose versus low glucose), ****p = 6.7e-6 (LF-BAP [low glucose] versus SF-BAP), ****p = 2.6e-9 (LF-BAP [high glucose] versus SF-BAP). All statistical analysis was conducted by a one-way ANOVA with two-sided Sidak's post hoc p-value adjustment for multiple comparisons. Source data are provided as a Source Data file.

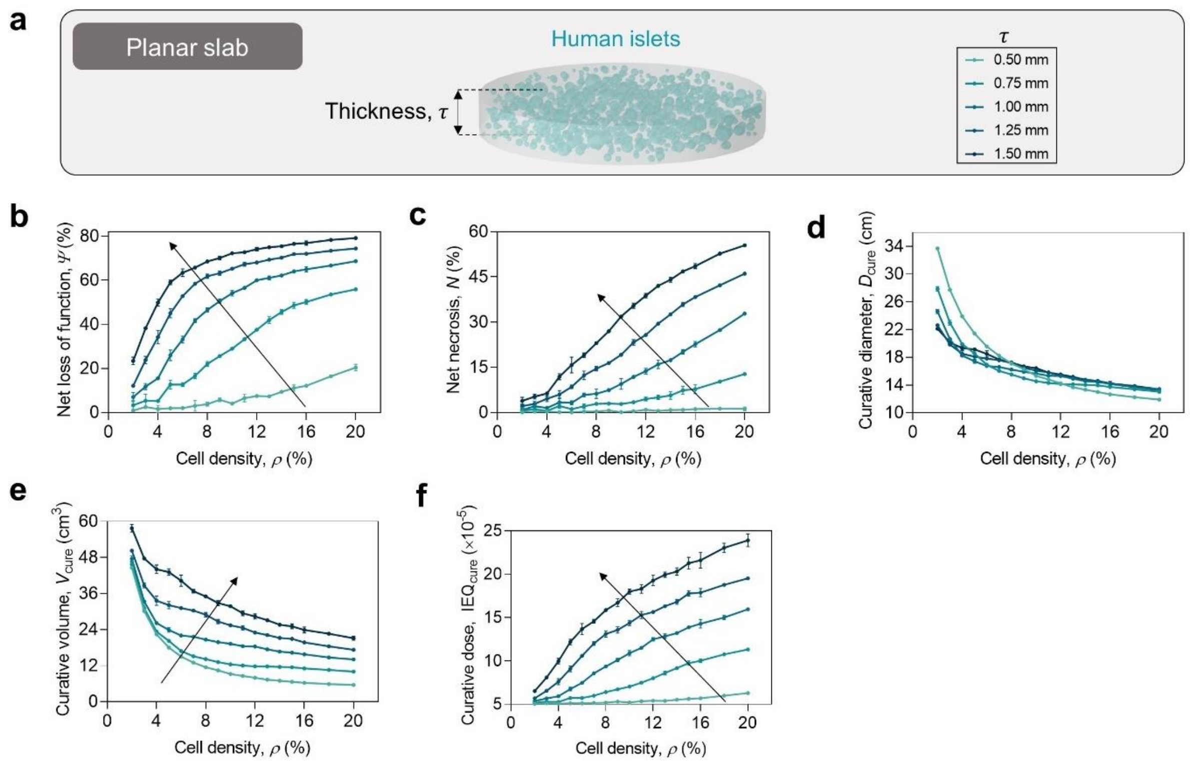

Supplementary Fig. 23 Planar slab optimizations. a Schematic describing the BAP system and figure legend. **b–f** Net loss of function (**b**), net necrosis (**c**), curative device diameter, D_{cure} (**d**), curative volume, V_{cure} (**e**), and curative dose, IEQ_{cure} (**f**) versus cell density, ρ, at varying slab thicknesses, τ. Arrows show general outcome parameter relationships with increasing slab thickness. Data shown represent mean ± SD from three replicates at each combination of ρ and τ. Source data are provided as a Source Data file.

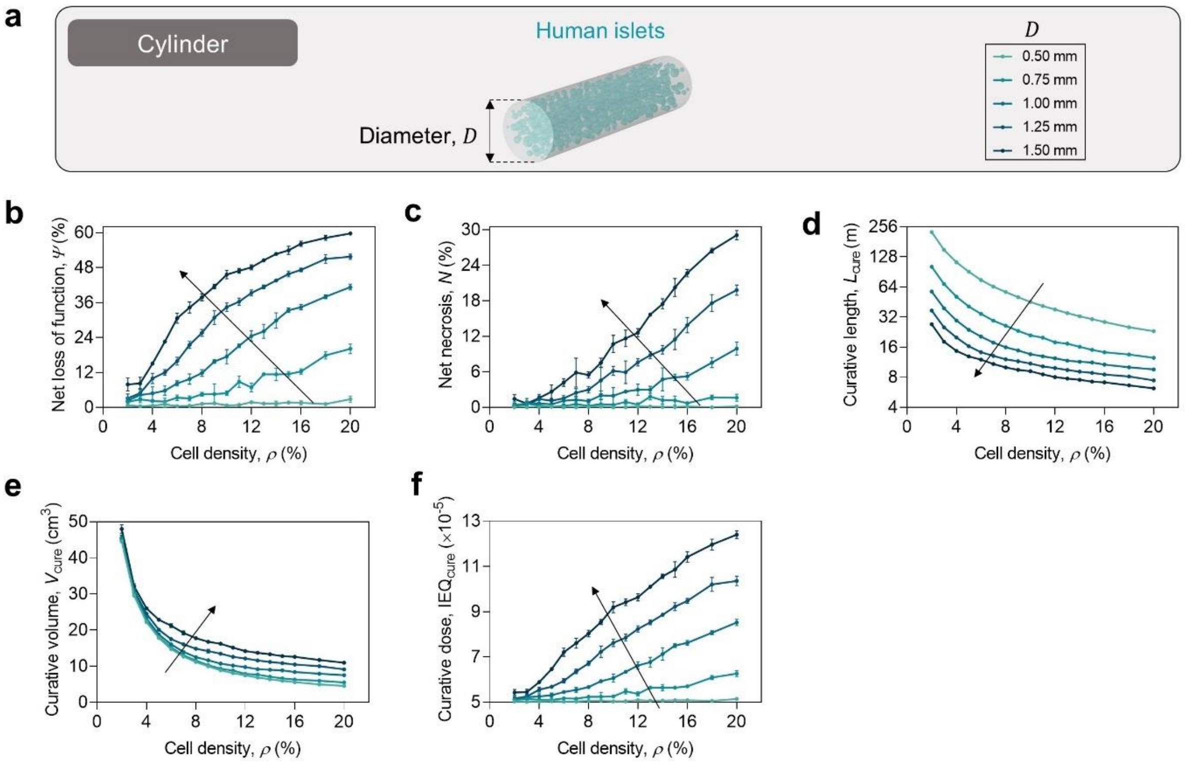

Supplementary Fig. 24 Cylinder optimizations. a Schematic describing the BAP system and figure legend. **b–f** Net loss of function (**b**), net necrosis (**c**), curative device length, L_{cure} (**d**), curative volume, V_{cure} (**e**), and curative dose, IEQ_{cure} (**f**) versus cell density, ρ, at varying cylinder diameters, D. Arrows show general outcome parameter relationships with increasing cylinder diameter. Data shown represent mean ± SD from three replicates at each combination of ρ and D. Source data are provided as a Source Data file.

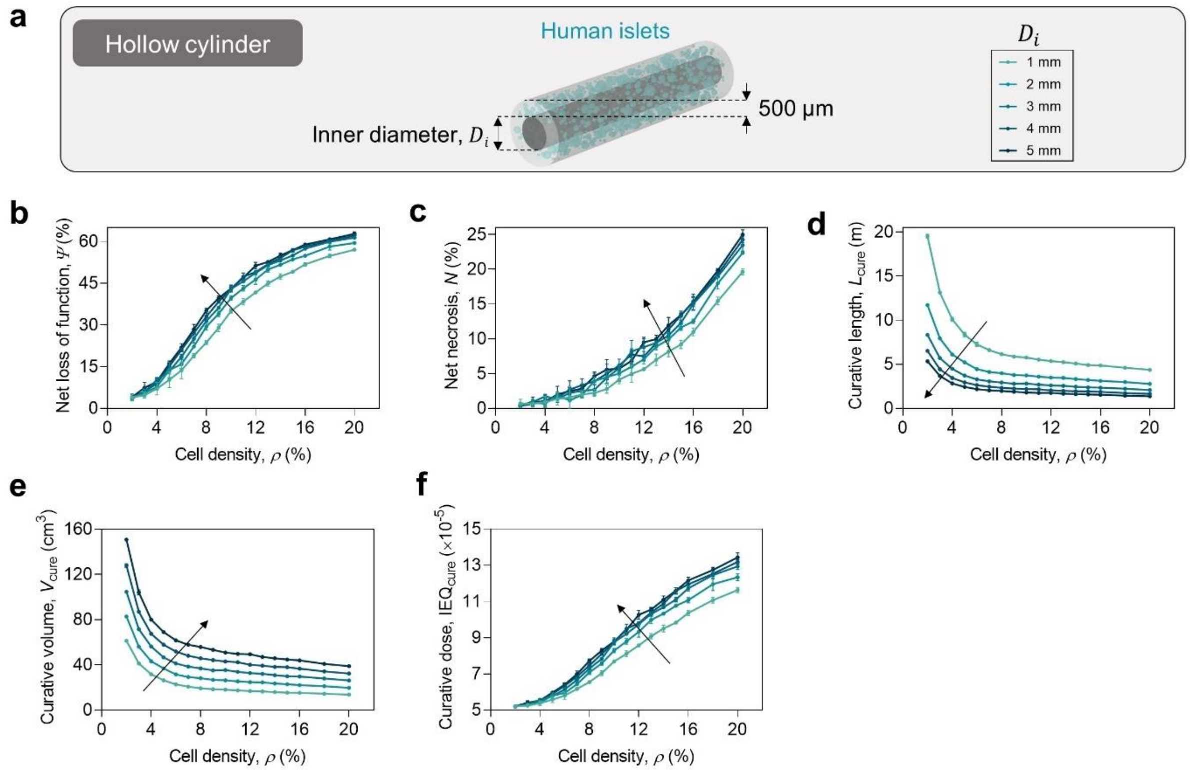

Supplementary Fig. 25 Hollow cylinder optimizations. a Schematic describing the BAP system and figure legend. **b–f** Net loss of function (**b**), net necrosis (**c**), curative device length, L_{cure} (**d**), curative volume, V_{cure} (**e**), and curative dose, IEQcure (**f**) versus cell density, ρ, at varying inner diameters, D_i. Arrows show general outcome parameter relationships with increasing inner diameter. Data shown represent mean ± SD from three replicates at each combination of ρ and D_i. Source data are provided as a Source Data file.

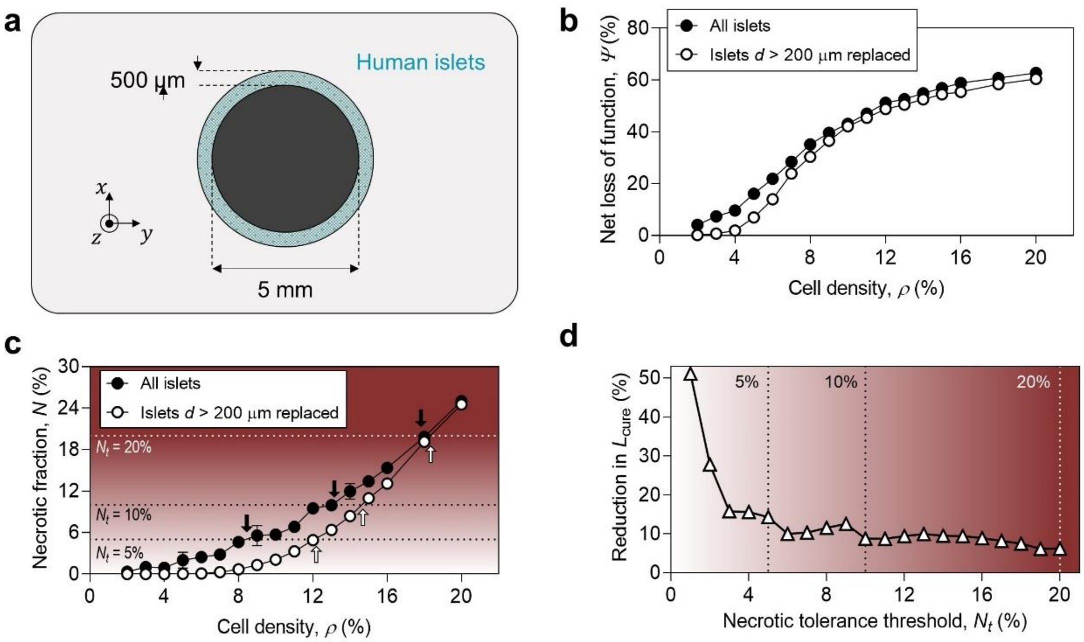

Supplementary Fig. 26 Removing the largest islets reduces required curative length. a Schematic describing the BAP system considered to explore the benefit of removing large islets on the curative length. **b** Net loss of function in the model BAP device at variable cell density when all islets were considered (n = 3) versus when islets d > 200 μm were removed (n = 3). A modest benefit is observed from removing the largest islets at all densities. **c** Net necrotic fraction versus cell density when all islets were considered (n = 3) or when islets d > 200 μm were removed (n = 3). Dashed lines indicate main necrotic tolerances, N_t, applied in the main text. Black and white arrows indicate density at which the "all islets" or "islets d > 200 μm removed" group intersected with each N_t, respectively. At any given necrotic tolerance, devices may incorporate higher cell densities if the largest islets are removed. The combination of the effects described above allows for a notable reduction in the required curative length, L_{cure}. Data are presented as mean $\pm$ SD. **d** Percent reduction in L_{cure} achievable from removing the largest islets at variable necrotic tolerance (N_t). Linear interpolation from the means of three replicates from both groups were used to determine the respective ρ for $\{N_t | N_t \in \mathbb{Z}, 1 \leq N_t \leq 20\}$. If conservative assumptions are made regarding the acceptable level of necrosis in such devices, a significant reduction in the size of a curative device may be achieved by removing the largest islets. Source data are provided as a Source Data file.

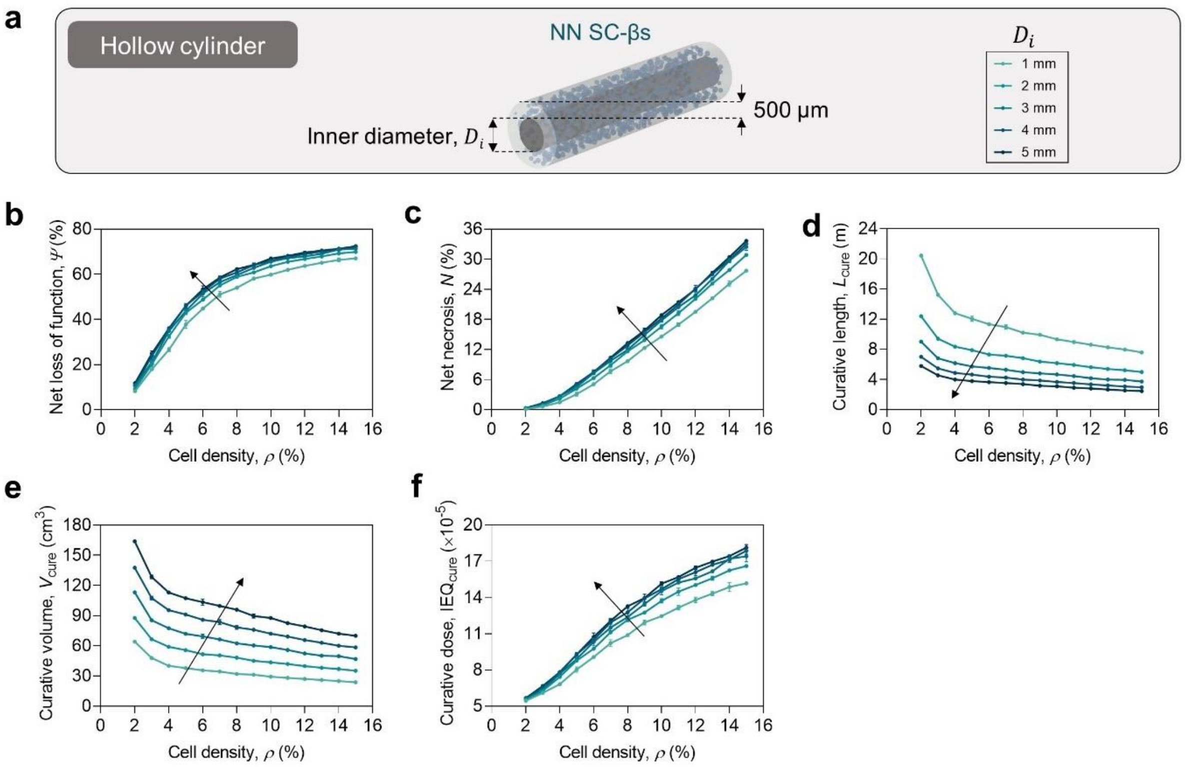

Supplementary Fig. 27 Hollow cylinder optimizations with NN SC-βs. a Schematic describing the BAP system and figure legend. **b–f** Net loss of function (**b**), net necrosis (**c**), curative device length, L_{cure} (**d**), curative volume, V_{cure} (**e**), and curative dose, IEQ_{cure} (**f**) versus cell density, ρ, at varying inner diameters, D_i. Arrows show general outcome parameter relationships with increasing inner diameter. Data shown represent mean ± SD from three replicates at each combination of ρ and D_i. Source data are provided as a Source Data file.

	SHARP	SHARP-ML
**System requirements**	Programming software and finite element solver	Internet access or offline app
**Computational time/burden**	Minutes to hours on powerful computer	Seconds, low
**Versatility**	Virtually any conceivable cell delivery system	Limited to general systems

Supplementary Fig. 28 Advantages and disadvantages of SHARP and SHARP-ML. SHARP, which utilizes the stochastic finite element method, requires proprietary software and a powerful computational machine but can simulate a versatile range of cell delivery systems, whereas SHARP-ML only requires internet access and a low computational burden (once trained), however it is, at present, limited to general systems similar to those already simulated on SHARP though it can feasibly be trained to a more versatile set of systems.

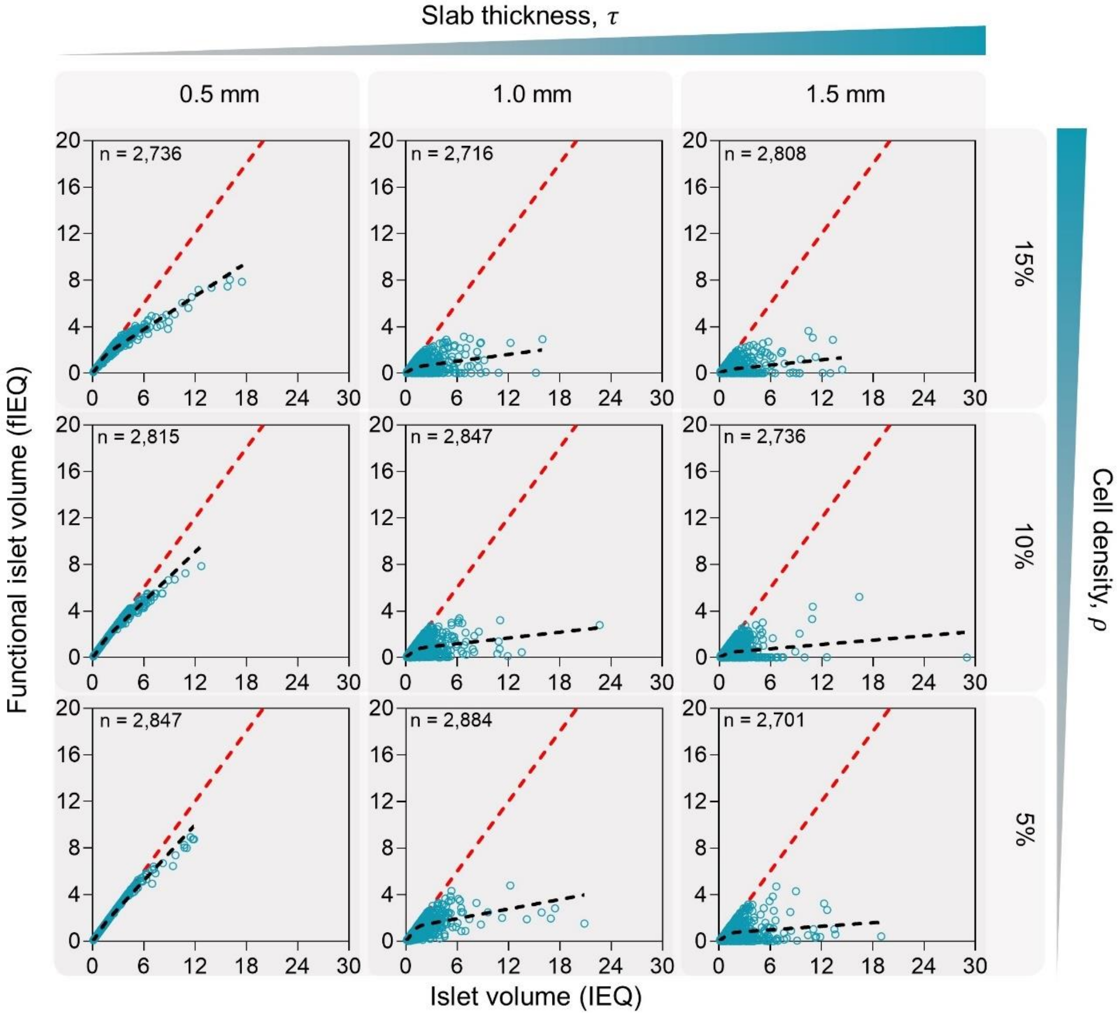

Supplementary Fig. 29 Functional islet equivalent volume is dependent on islet size and BAP system parameters. Islet volume, IEQ, versus functional islet volume, fIEQ, for 9 planar slab encapsulation systems defined by all combinations of cell density, ρ = {5%, 10%, 15%}, and slab thickness, τ = {0.5 mm, 1.0 mm, 1.5 mm}. Open circles represent values for individual cell clusters collected from three iterations of each tested combination of ρ and τ (n represents the total number of simulated islets for each input variable combination); the red dashed line represents line of identity, and the black dashed line represents a spline fit. Source data are provided as a Source Data file.

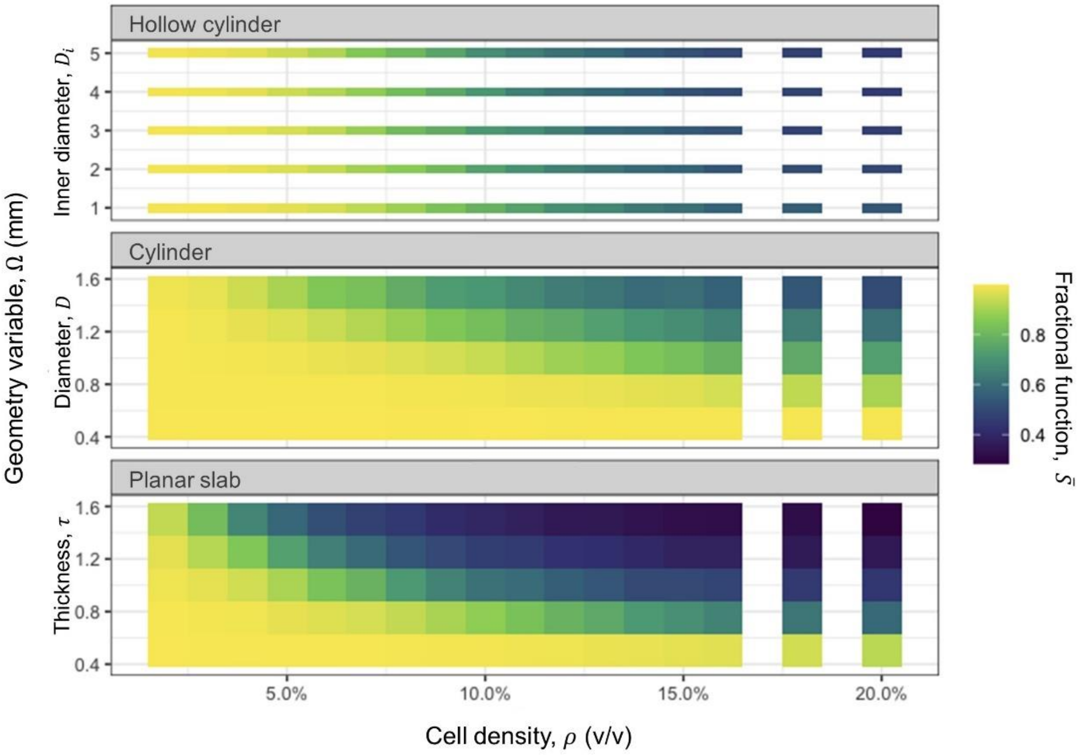

Supplementary Fig. 30 Ensemble machine learning model (SHARP-ML) training data. Heat map of net fractional function, $\bar{S}$ (i.e., relative insulin secretion capacity) for all combinations of cell density, ρ, and geometry variable, Ω (i.e., slab thickness, τ, cylinder diameter, D, or hollow cylinder inner diameter, D_i) calculated by SHARP in the optimization study (Fig. 5). The color of each square represents the mean $\bar{S}$ from three replications. SHARP-ML was trained to κ_{fIEQ} data extracted from each combination of cell density, and geometry variable. Source data are provided as a Source Data file.

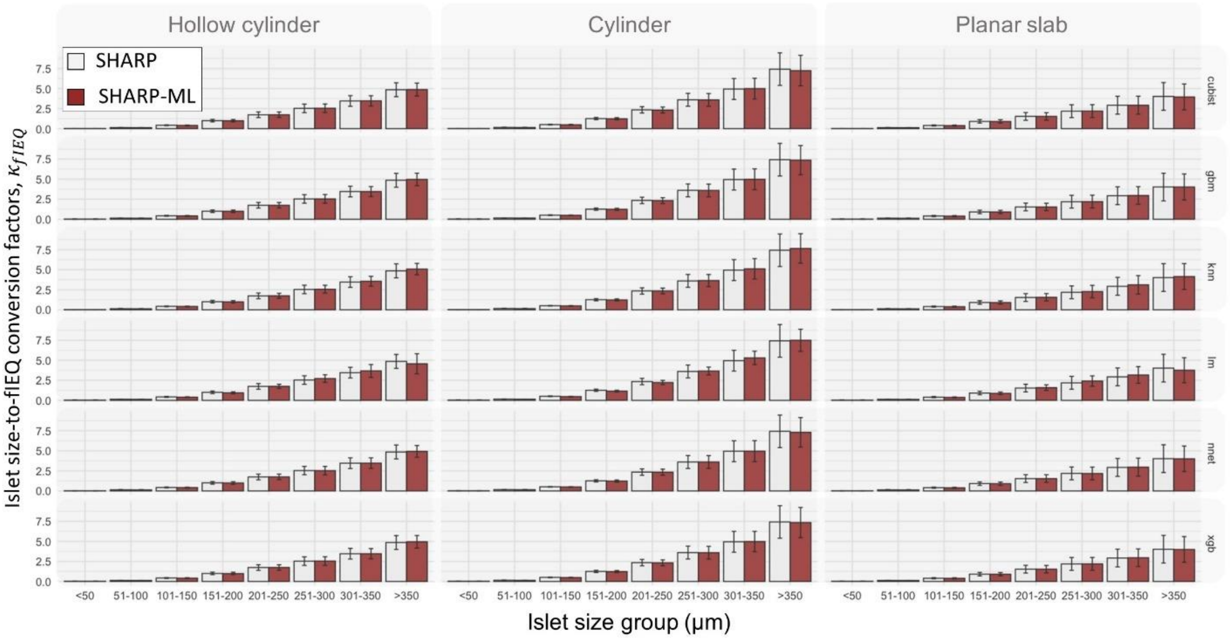

Supplementary Fig. 31 Out-of-sample estimates for each machine learning model. SHARP calculations (white; hollow cylinder: n = 210 iterations; cylinder: 135 iterations; planar slab: n = 165 iterations) versus SHARP-ML predictions (red; n = 18 model observations summarized from > 2 $\times 10^7$ observations) of each geometry's aggregated (i.e., all combinations of cell density and geometry variable) islet size-to-fIEQ conversion factors, κ_{fIEQ}, for each 50 μm-width bin, for each constituent model of the machine learning ensemble: (top to bottom) cubist, LightBGM (gbm), K-nearest neighbors (knn), linear model (lm), neural network (nnet), and XGBoost (xgb). Data shown represent mean ± SD. Source data are provided as a Source Data file.

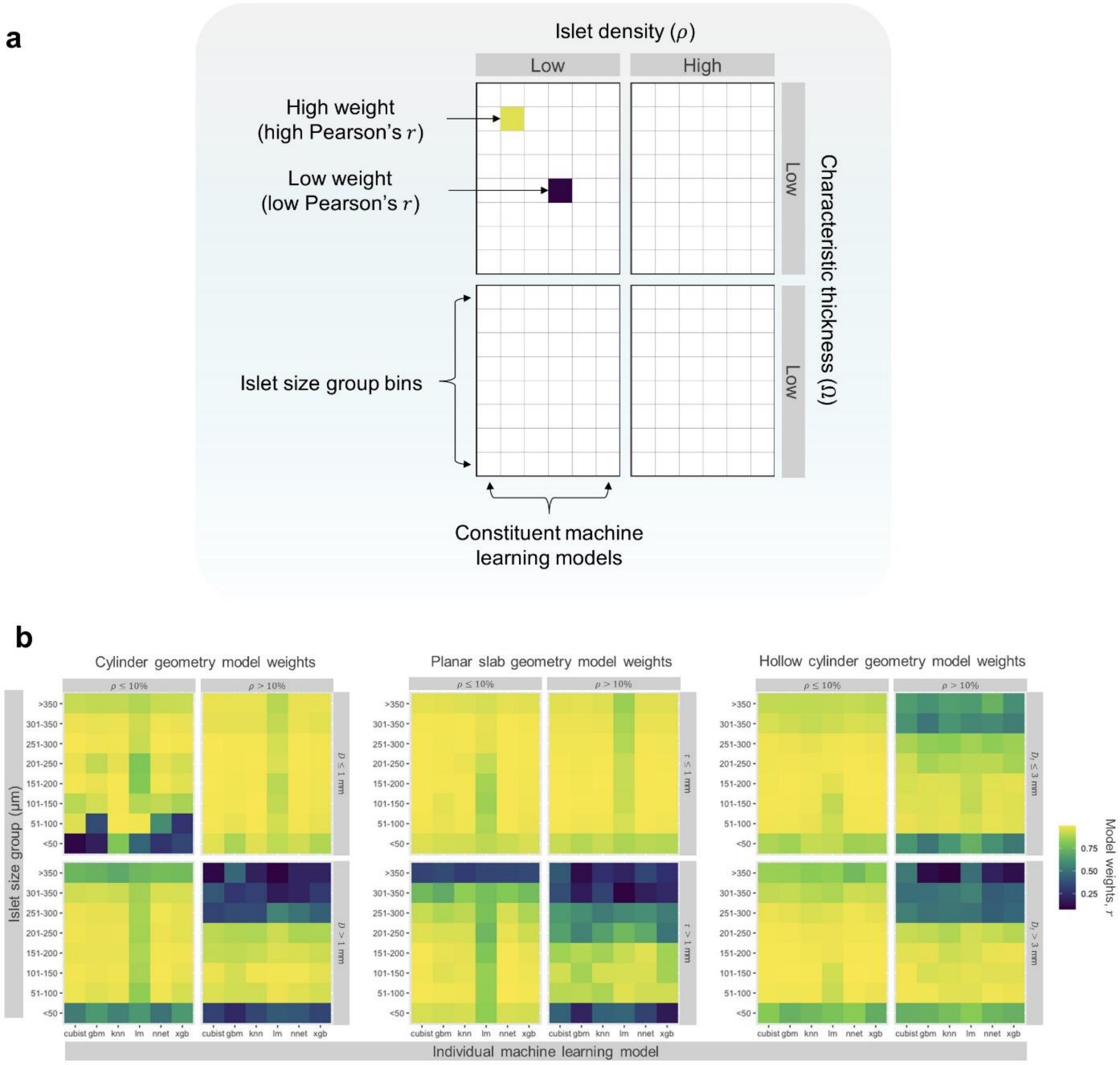

Supplementary Fig. 32 Constituent machine learning model weights. a Guide for interpreting model weight charts in **b**: performance of the constituent machine learning models, defined by the Pearson's correlation coefficient (r) is shown in four major subgroups of the parameter space, for each islet size group. The Pearson's r is used as the weights of each underlying model's contribution to the ensemble in the relevant regions of the parameter space. **b** Absolute weights (Pearson's r) for each machine learning model, geometry, and combinations of cell density (ρ) and geometry variable (cylinder diameter, D, planar slab thickness, τ, or hollow cylinder inner diameter, D_i) grouped by their relationship to the median values for each geometry. Source data are provided as a Source Data file.

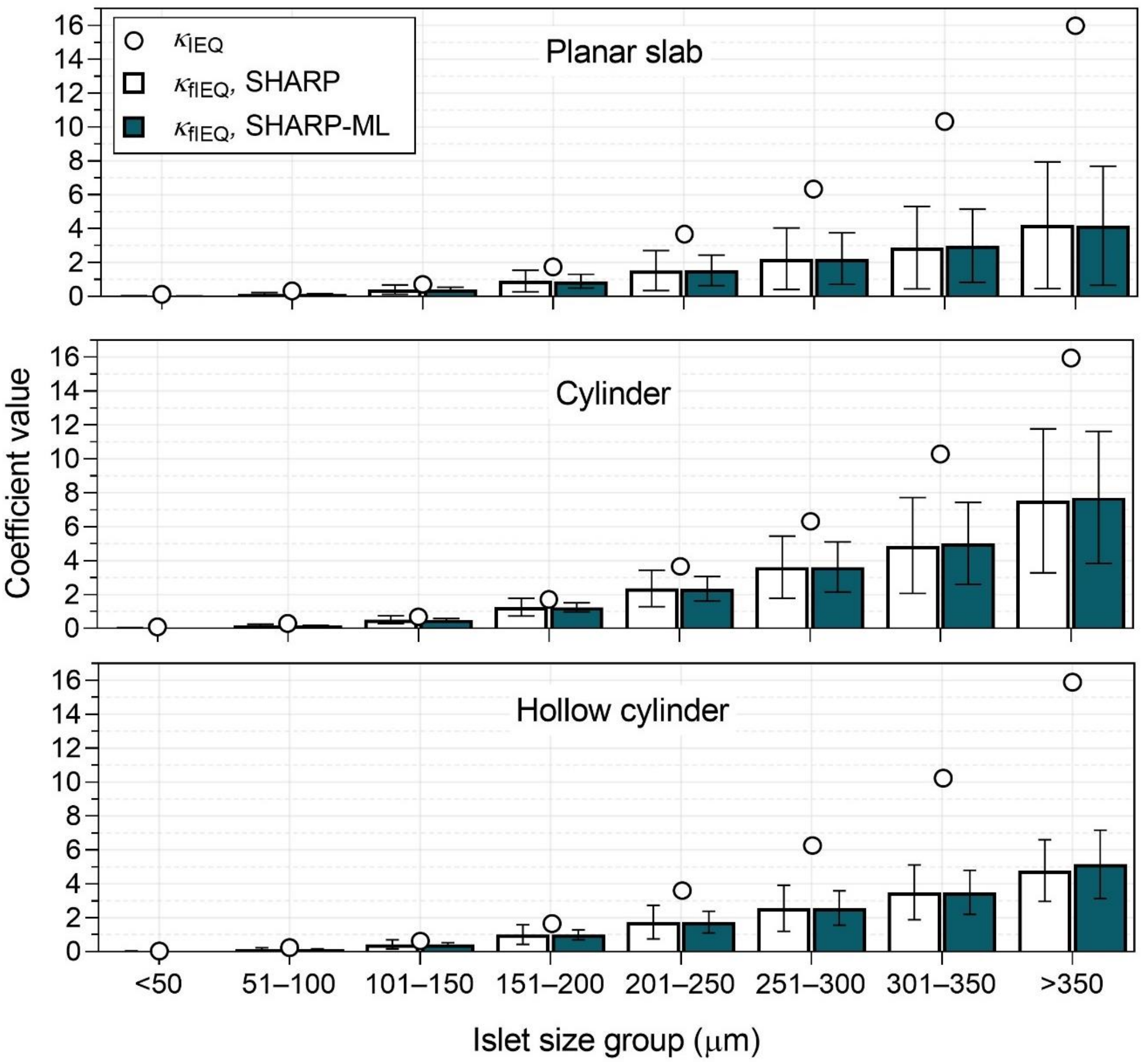

Supplementary Fig. 33 Ensemble machine learning model predictions. Conventional conversion coefficients, κ_{IEQ} (open circles), and function-adjusted conversion coefficients, κ_{fIEQ}, as predicted by SHARP (white bars; n = 150 iterations) and SHARP-ML (teal bars; n = 18 model predictions summarized from > 2×10^7 observations) for each geometry's aggregated data from all combinations of cell density (ρ) and geometry variable (Ω). SHARP-ML predictions closely matched the mean value and distribution of data for all groups of all geometries. Data for both κ_{fIEQ} results (teal and white bars) represent mean ± SD. Source data are provided as a Source Data file.

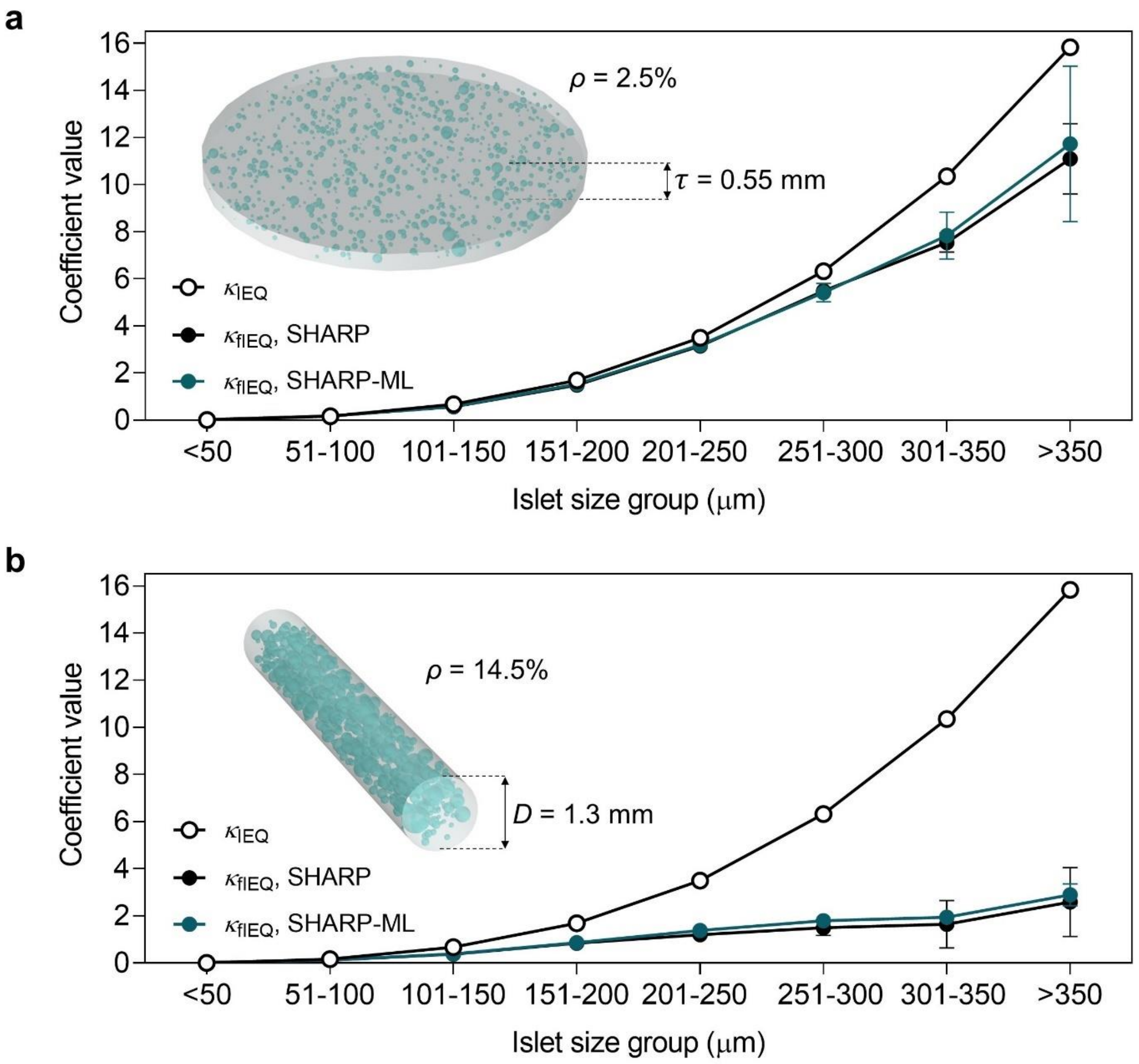

Supplementary Fig. 34 SHARP-ML predicts islet size-to-fIEQ conversion coefficients in two hypothetical BAP devices. a, b Conventional conversion coefficients, κ_{IEQ}, and function-adjusted conversion coefficients, κ_{fIEQ}, as predicted by SHARP-ML (n = 18 model predictions summarized from > 2 × 10^7 observations) and calculated by SHARP (n = 20 iterations) for two hypothetical encapsulation systems. Plots are visual representations of the values provided in Fig. 6b. Data are presented as mean ± SD. Source data are provided as a Source Data file.

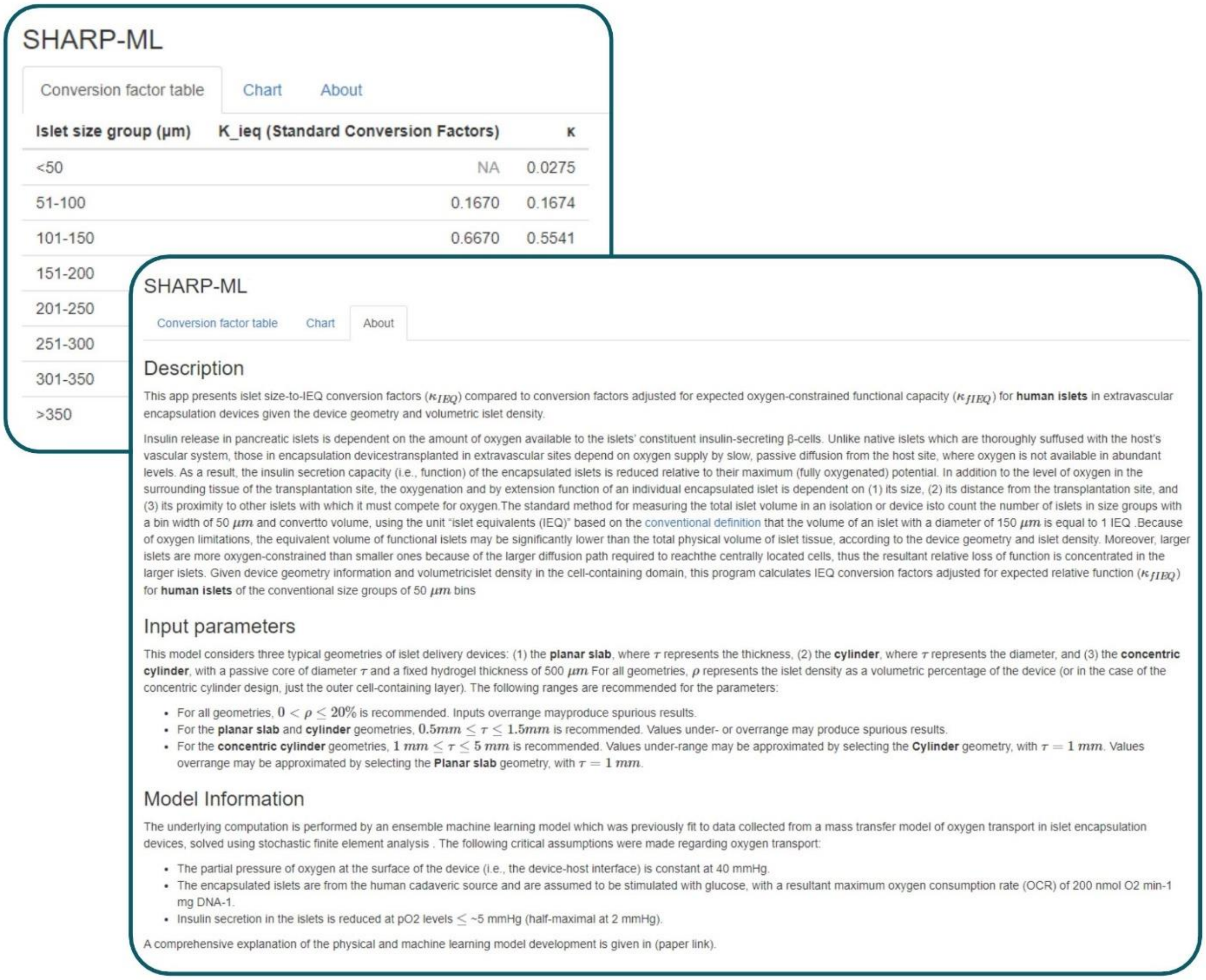

Supplementary Fig. 35 Screenshots from the SHARP-ML web application. Additional screenshots from the SHARP-ML web application showing the “Conversion factor table” and “About” tabs.

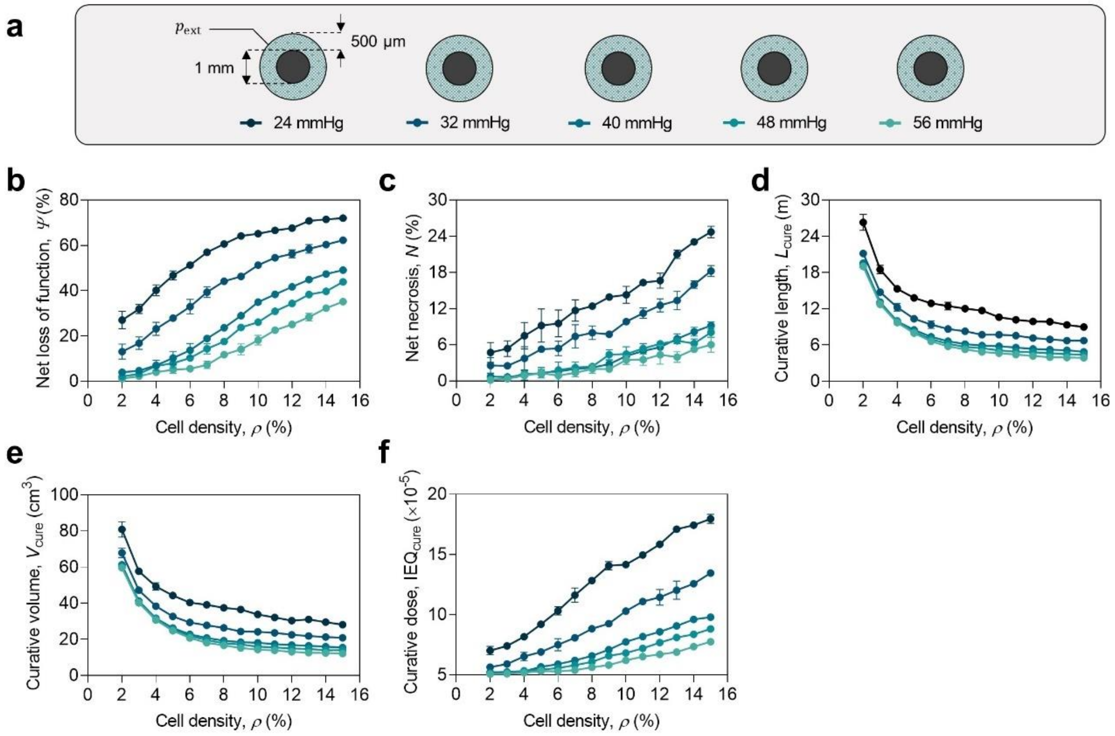

Supplementary Fig. 36 Effects of variable boundary oxygen tension on device performance and curative feasibility. a Schematic illustrating the analyzed BAP system (containing human islets): the performance of a hollow cylinder system with a passive core diameter of 1 mm and hydrogel thickness of 500 µm was analyzed under boundary oxygen tensions (p_{ext}) of 24, 32, 40, 48, and 56 mmHg. **b–f** Net loss of function, Ψ (**b**), net necrotic percentage, N (**c**), and expected curative length, L_{cure} (**d**), curative volume, V_{cure} (**e**), and curative dose, IEQ$_{\text{cure}}$ (**f**). Data shown represent mean ± SD from three replicates at each combination of ρ and p_{ext}. Source data are provided as a Source Data file.

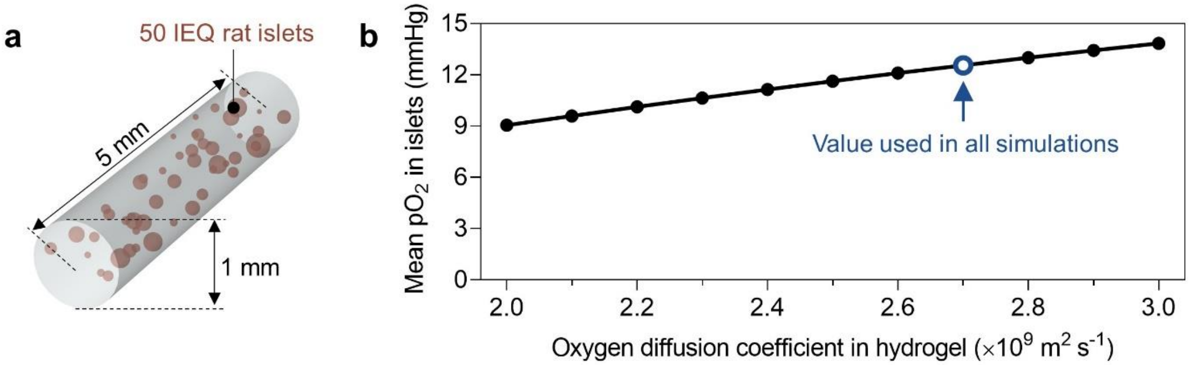

Supplementary Fig. 37 Model sensitivity to the diffusion coefficient of oxygen in hydrogel. a, b Schematic of the test construct (**a**) and mean pO_2 in the islets of the test construct versus the implemented value of the diffusion coefficient of oxygen in hydrogel ($D_{O_2,h}$) (**b**). Source data are provided as a Source Data file.

SI References

1 Buchwald, P. *et al.* Quantitative assessment of islet cell products: Estimating the accuracy of the existing protocol and accounting for islet size distribution. *Cell Transplant.* **18**, 1223-1235, (2009).

2 Buchwald, P. A local glucose-and oxygen concentration-based insulin secretion model for pancreatic islets. *Theor. Biol. Med. Model.* **8**, 20, (2011).

3 Buchwald, P., Tamayo‐Garcia, A., Manzoli, V., Tomei, A. A. & Stabler, C. L. Glucose‐stimulated insulin release: Parallel perifusion studies of free and hydrogel encapsulated human pancreatic islets. *Biotechnol. Bioeng.* **115**, 232-245, (2018).

4 Buchwald, P. Fem-based oxygen consumption and cell viability models for avascular pancreatic islets. *Theor. Biol. Med. Model.* **6**, 5, (2009).

5 Suszynski, T. M., Avgoustiniatos, E. S. & Papas, K. K. Oxygenation of the intraportally transplanted pancreatic islet. *J. Diabetes Res.* **2016**, 7625947, (2016).

6 Avgoustiniatos, E. S. *Oxygen diffusion limitations in pancreatic islet culture and immunoisolation* Doctor of Philosophy thesis, Massachusetts Institute of Technology, (2001).

7 Dulong, J.-J. & Legallais, C. A theoretical study of oxygen transfer including necrosis for the design of a bioartificial pancreas. *Biotechnol. Bioeng.* **96**, 990-998, (2006).

8 Dionne, K. E., Colton, C. K. & Yarmush, M. L. Effect of hypoxia on insulin secretion by isolated rat and canine islets of langerhans. *Diabetes* **42**, 12-21, (1993).

9 Ricordi, C. *et al.* Islet isolation assessment in man and large animaFls. *Acta Diabetol. Lat.* **27**, 185-195, (1990).

10 McCall, M. & Shapiro, A. M. J. Update on islet transplantation. *Cold Spring Harb. Perspect. Med.* **2**, a007823-a007823, (2012).

11 Li, X., Meng, Q. & Zhang, L. The fate of allogeneic pancreatic islets following intraportal transplantation: Challenges and solutions. *Journal of Immunology Research* **2018**, 2424586, (2018).

12 Biarnés, M. *et al.* Beta-cell death and mass in syngeneically transplanted islets exposed to short- and long-term hyperglycemia. *Diabetes* **51**, 66-72, (2002).

13 Ernst, A. U., Wang, L.-H. & Ma, M. Islet encapsulation. *J. Mater. Chem. B* **6**, 6705-6722, (2018).

14 Fuchs, S. *et al.* Hydrogels in emerging technologies for type 1 diabetes. *Chem. Rev.* **121**, 11458-11526, (2021).

15 Lewis, A. S. *Eliminating oxygen supply limitations for transplanted microencapsulated islets in the treatment of type 1 diabetes* Doctor of Philosophy thesis, Massachusetts Institute of Technology, (2008).

16 Mehmetoglu, Ü., Ateş, S. & Berber, R. Oxygen diffusivity in calcium alginate gel beads containing gluconobacter suboxydans. *Artif. Cells Blood Substit. Immobil. Biotechnol.* **24**, 91-106, (1996).

17 Kjeldsen, P. Evaluation of gas diffusion through plastic materials used in experimental and sampling equipment. *Water Res.* **27**, 121-131, (1993).

18 Avgoustiniatos, E. S. & Colton, C. K. Effect of external oxygen mass transfer resistances on viability of immunoisolated tissue. *Ann. N. Y. Acad. Sci.* **831**, 145-166, (1997).

19 Einstein, S. A., Weegman, B. P., Firpo, M. T., Papas, K. K. & Garwood, M. Development and validation of noninvasive magnetic resonance relaxometry for the in vivo assessment of tissue-engineered graft oxygenation. *Tissue Eng. Part C* **22**, 1009-1017, (2016).

20 Whitney, J. D., Stotts, N. A., Goodson, W. H., 3rd & Janson-Bjerklie, S. The effects of activity and bed rest on tissue oxygen tension, perfusion, and plasma volume. *Nurs. Res.* **42**, 349-355, (1993).

21 Shewry, M. C. & Wynn, H. P. Maximum entropy sampling. *Journal of Applied Statistics* **14**, 165-170, (1987).

22 Kuhn, M. & Wickham, H. Tidymodels: A collection of packages for modeling and machine learning using tidyverse principles. https://www.tidymodels.org, (2020).

23 Wang, W., Winlove, C. P. & Michel, C. C. Oxygen partial pressure in outer layers of skin of human finger nail folds. *J. Physiol.* **549**, 855-863, (2003).

24 Bochenek, M. A. *et al.* Alginate encapsulation as long-term immune protection of allogeneic pancreatic islet cells transplanted into the omental bursa of macaques. *Nat. Biomed. Eng.* **2**, 810-821, (2018).

25 Mellström, Å., Hartmann, M., Jedlinska, B. & Jönsson, K. Effect of hyperoxia and hypoxia on subcutaneous tissue gases and ph. *Eur. Surg. Res.* **31**, 333-339, (1999).

26 Vériter, S. *et al.* The impact of hyperglycemia and the presence of encapsulated islets on oxygenation within a bioartificial pancreas in the presence of mesenchymal stem cells in a diabetic wistar rat model. *Biomaterials* **32**, 5945-5956, (2011).

27 Vériter, S. *et al.* In vivo selection of biocompatible alginates for islet encapsulation and subcutaneous transplantation. *Tissue Eng Part A* **16**, 1503-1513, (2010).

28 Komatsu, H. *et al.* Posttransplant oxygen inhalation improves the outcome of subcutaneous islet transplantation: A promising clinical alternative to the conventional intrahepatic site. *Am. J. Transplantation* **18**, 832-842, (2017).

29 Sawyer, R. G., Spengler, M. D., Adams, R. B. & Pruett, T. L. The peritoneal environment during infection. The effect of monomicrobial and polymicrobial bacteria on po2 and ph. *Ann. Surg.* **213**, 253-260, (1991).

30 Lutz, J. *et al.* Measurement of oxygen tensions in the abdominal cavity and in the skeletal muscle using 19f-mri of neat pfc droplets. *Adv. Exp. Med. Biol.* **428**, 569-572, (1997).

31 Nöth, U. *et al.* 19f-mri in vivo determination of the partial oxygen pressure in perfluorocarbon-loaded alginate capsules implanted into the peritoneal cavity and different tissues. *Magn. Reson. Med.* **42**, 1039-1047, (1999).

32 Bourdel, N. *et al.* Peritoneal tissue-oxygen tension during a carbon dioxide pneumoperitoneum in a mouse laparoscopic model with controlled respiratory support. *Hum. Reprod.* **22**, 1149-1155, (2007).

33 Colton, C. K. *et al.* in *Cellular transplantation* (eds Craig Halberstadt & Dwaine Emerich) 85-133 (Academic Press, 2007).

34 Doliba, N. M. *et al.* Cholinergic regulation of fuel-induced hormone secretion and respiration of sur1-/- mouse islets. *Am. J. Physiol. Endocrinol. Metab.* **291**, E525-535, (2006).

35 Avgoustiniatos, E. S., Dionne, K. E., Wilson, D. F., Yarmush, M. L. & Colton, C. K. Measurements of the effective diffusion coefficient of oxygen in pancreatic islets. *Ind. Eng. Chem. Res.* **46**, 6157-6163, (2007).

36 Weegman, B. P. *et al.* Plasticity and aggregation of juvenile porcine islets in modified culture: Preliminary observations. *Cell Transplant.* **25**, 1763-1775, (2016).

37 Papas, K. K., Pisania, A., Wu, H., Weir, G. C. & Colton, C. K. A stirred microchamber for oxygen consumption rate measurements with pancreatic islets. *Biotechnol. Bioeng.* **98**, 1071-1082, (2007).

38 Jo, J., Choi, M. Y. & Koh, D.-S. Size distribution of mouse langerhans islets. *Biophys. J.* **93**, 2655-2666, (2007).
