# Supplementary material for: A predictive computational platform for optimizing the design of bioartificial pancreas devices
Source: Nat Commun. 2022 Oct 13;13:6031. doi: 10.1038/s41467-022-33760-5 (PMC9561707; doi:10.1038/s41467-022-33760-5)
Supplement: Supplementary file 3 — Description of additional supplementary files [file 41467_2022_33760_MOESM3_ESM.pdf]

# Description of additional supplementary files

Supplementary Data 1: SHARP-ML training grid and diameter counts.

Supplementary Data 2: SHARP-ML summary of general observations.

Supplementary Data 3: SHARP-ML constituent model hyperparameters.

Supplementary Data 4: SHARP-ML model predictions across the overall parameter space.

Supplementary Data 5: Hyperparameter tuning function and final model specifications for SHARPML.
