# Supplementary material for: A predictive computational platform for optimizing the design of bioartificial pancreas devices
Source: Nat Commun. 2022 Oct 13;13:6031. doi: 10.1038/s41467-022-33760-5 (PMC9561707; doi:10.1038/s41467-022-33760-5)
Supplement: Supplementary file 8 — Supplementary Data 5 [file 41467_2022_33760_MOESM8_ESM.pdf]

model_specifications_sharp_ml

Scott Worland

8/4/2022

Hyper parameter tuning function

The hyperparameters for each model were tuned using a hyperparameter tuning workflow set created by the R function below.

```
#' build workflow set of models
#' @param model_rec recipe: preprocessing steps
build_workflow_set <- function(model_rec){

  # linear model spec
  lm_spec <-
    linear_reg() %>%
    set_engine('lm') %>%
    set_mode('regression')

  # lightGBM spec
  gbm_spec <-
    boost_tree(mtry = tune(),
               trees = tune(),
               min_n = tune(),
               tree_depth = tune(),
               loss_reduction = tune(),
               learn_rate = tune(),
               sample_size = 0.75) %>%
    set_engine("lightgbm") %>%
    set_mode("regression")


  # xgboost spec
  xgb_spec <-
    boost_tree(mtry = tune(),
               trees = tune(),
               min_n = tune(),
               tree_depth = tune(),
               loss_reduction = tune(),
               learn_rate = tune(),
               sample_size = 0.75) %>%
    set_engine("xgboost") %>%
    set_mode("regression")

  # cubist spec
```

```r
cubist_spec <-
  cubist_rules(committees = tune(),
               neighbors = tune(),
               max_rules = tune()) %>%
  set_engine('Cubist') %>%
  set_mode("regression")

# neural net spec
nnet_spec <-
  mlp(
    hidden_units = tune(),
    penalty = tune(),
    epochs = 300,
    activation = 'relu') %>%
  set_engine('nnet') %>%
  set_mode("regression")

# knn spec
knn_spec <-
  nearest_neighbor(
    neighbors = tune(),
    weight_func = tune(),
    dist_power = tune()) %>%
  set_engine('kknn') %>%
  set_mode("regression")

## update default parameter ranges ----

# update cubist params
cubist_params <-
  cubist_spec %>%
  parameters() %>%
  update(
    neighbors = neighbors(range=c(0,5)),
    max_rules = max_rules(c(1,500))
  )

# update gbm params
gbm_params <-
  gbm_spec %>%
  parameters() %>%
  update(
    mtry = mtry(range(c(1,11))),
    trees = trees(range=c(50,1500)),
    min_n = min_n(range=c(5,100))
  )

# update xgb params
xgb_params <-
  xgb_spec %>%
  parameters() %>%
  update(
    mtry = mtry(range(c(1,11))),
```

```
      trees = trees(range=c(50,1500)),
      min_n = min_n(range=c(5,100))
    )

  # update nnet params
  nnet_params <-
    nnet_spec %>%
    parameters() %>%
    update(
      hidden_units = hidden_units(range=c(5,25))
    )

  # update knn params
  knn_params <-
    knn_spec %>%
    parameters() %>%
    update(
      weight_func = weight_func(values=c('gaussian','rectangular'))
    )

  ## Create workflow set ----

  # model spec list
  model_specs <- list(
    lm = lm_spec,
    nnet = nnet_spec,
    knn = knn_spec,
    xgb = xgb_spec,
    gbm = gbm_spec,
    cubist = cubist_spec
  )

  # workflow set
  wkflow_set <- workflow_set(
    preproc = list(base_rec = model_rec),
    models = model_specs,
    cross = TRUE) %>%
    mutate(wflow_id = names(model_specs)) %>%
    option_add(param_info = cubist_params, id = "cubist") %>%
    option_add(param_info = gbm_params, id = "gbm") %>%
    option_add(param_info = nnet_params, id = 'nnet') %>%
    option_add(param_info = xgb_params, id = 'xgb') %>%
    option_add(param_info = knn_params, id = 'knn')

  return(wkflow_set)

}
```

Post tuning model specifications for the planar slab geometry

Linear model (LM)

```
## parsnip model object
##
## Fit time:  1ms
##
## Call:
## stats::lm(formula = ..y ~ ., data = data)
##
## Coefficients:
## (Intercept)          tau          rho     diameter
##      0.5439      -0.1879      -0.1228      -0.2059
```

Cubist model (cubist)

```
## Cubist Model Specification (regression)
##
## Main Arguments:
##   committees = 90
##   neighbors = 2
##   max_rules = 467
##
## Computational engine: Cubist
```

K-nearest neighbors (KNN)

```
## K-Nearest Neighbor Model Specification (regression)
##
## Main Arguments:
##   neighbors = 5
##   weight_func = gaussian
##   dist_power = 1.31076544504799
##
## Computational engine: kknn
```

Neural network (NN)

```
## Single Layer Neural Network Specification (regression)
##
## Main Arguments:
##   hidden_units = 19
##   penalty = 4.40483411134666e-06
##   epochs = 300
##   activation = relu
##
## Computational engine: nnet
```

Light Gradient boosting machine (GBM)

```
## Boosted Tree Model Specification (regression)
##
## Main Arguments:
##   mtry = 4
##   trees = 933
##   min_n = 30
##   tree_depth = 14
##   learn_rate = 0.0818937796439461
##   loss_reduction = 3.50898151760879e-06
##   sample_size = 0.75
##
## Computational engine: lightgbm
```

Extreme gradient boosting (XGB)

```
## Boosted Tree Model Specification (regression)
##
## Main Arguments:
##   mtry = 4
##   trees = 933
##   min_n = 30
##   tree_depth = 14
##   learn_rate = 0.0818937796439461
##   loss_reduction = 3.50898151760879e-06
##   sample_size = 0.75
##
## Computational engine: xgboost
```

Post tuning model specifications for cylinder geometry

Linear model (LM)

```
## parsnip model object
##
## Fit time:  1ms
##
## Call:
## stats::lm(formula = ..y ~ ., data = data)
##
## Coefficients:
## (Intercept)          tau          rho     diameter
##     0.75180     -0.14325     -0.09294     -0.17898
```

Cubist model (cubist)

```
## Cubist Model Specification (regression)
##
## Main Arguments:
##   committees = 27
```

```
##   neighbors = 0
##   max_rules = 195
##
## Computational engine: Cubist
```

K-nearest neighbors (KNN)

```
## K-Nearest Neighbor Model Specification (regression)
##
## Main Arguments:
##   neighbors = 7
##   weight_func = gaussian
##   dist_power = 1.26145643531461
##
## Computational engine: kknn
```

Neural network (NN)

```
## Single Layer Neural Network Specification (regression)
##
## Main Arguments:
##   hidden_units = 12
##   penalty = 0.00200236036551463
##   epochs = 300
##   activation = relu
##
## Computational engine: nnet
```

Light Gradient boosting machine (GBM)

```
## Boosted Tree Model Specification (regression)
##
## Main Arguments:
##   mtry = 5
##   trees = 1135
##   min_n = 46
##   tree_depth = 10
##   learn_rate = 0.0548323184136669
##   loss_reduction = 1.06342275801344e-08
##   sample_size = 0.75
##
## Computational engine: lightgbm
```

Extreme gradient boosting (XGB)

```
## Boosted Tree Model Specification (regression)
##
## Main Arguments:
##   mtry = 5
##   trees = 1135
##   min_n = 46
```

```
##   tree_depth = 10
##   learn_rate = 0.0548323184136669
##   loss_reduction = 1.06342275801344e-08
##   sample_size = 0.75
##
## Computational engine: xgboost
```

Post tuning model specifications for annulus geometry

Linear model (LM)

```
## parsnip model object
##
## Fit time:  2ms
##
## Call:
## stats::lm(formula = ..y ~ ., data = data)
##
## Coefficients:
## (Intercept)          tau          rho     diameter
##     0.59690     -0.02247     -0.16078     -0.19488
```

Cubist model (cubist)

```
## Cubist Model Specification (regression)
##
## Main Arguments:
##   committees = 50
##   neighbors = 5
##   max_rules = 25
##
## Computational engine: Cubist
```

K-nearest neighbors (KNN)

```
## K-Nearest Neighbor Model Specification (regression)
##
## Main Arguments:
##   neighbors = 10
##   weight_func = gaussian
##   dist_power = 0.861960433675675
##
## Computational engine: kknn
```

Neural network (NN)

```
## Single Layer Neural Network Specification (regression)
##
## Main Arguments:
##   hidden_units = 6
```

```
##   penalty = 4.37645577119682e-10
##   epochs = 300
##   activation = relu
##
## Computational engine: nnet
```

Light Gradient boosting machine (GBM)

```
## Boosted Tree Model Specification (regression)
##
## Main Arguments:
##   mtry = 4
##   trees = 898
##   min_n = 33
##   tree_depth = 4
##   learn_rate = 0.0147132681907278
##   loss_reduction = 5.52674923383885e-07
##   sample_size = 0.75
##
## Computational engine: lightgbm
```

Extreme gradient boosting (XGB)

```
## Boosted Tree Model Specification (regression)
##
## Main Arguments:
##   mtry = 5
##   trees = 1441
##   min_n = 16
##   tree_depth = 4
##   learn_rate = 0.0313792505030693
##   loss_reduction = 2.24595078077267e-08
##   sample_size = 0.75
##
## Computational engine: xgboost
```
