# Supplementary material for: A predictive computational platform for optimizing the design of bioartificial pancreas devices
Source: Nat Commun. 2022 Oct 13;13:6031. doi: 10.1038/s41467-022-33760-5 (PMC9561707; doi:10.1038/s41467-022-33760-5)
Supplement: Supplementary file 9 — Reporting Summary [file 41467_2022_33760_MOESM9_ESM.pdf]

nature research

Corresponding author(s): Minglin Ma, Long-Hai Wang

Last updated by author(s): Sep 22, 2022

Reporting Summary

Nature Research wishes to improve the reproducibility of the work that we publish. This form provides structure for consistency and transparency in reporting. For further information on Nature Research policies, see our Editorial Policies and the Editorial Policy Checklist.

Statistics

For all statistical analyses, confirm that the following items are present in the figure legend, table legend, main text, or Methods section.

n/a	Confirmed	
☐	☒	The exact sample size (*n*) for each experimental group/condition, given as a discrete number and unit of measurement
☐	☒	A statement on whether measurements were taken from distinct samples or whether the same sample was measured repeatedly
☐	☒	The statistical test(s) used AND whether they are one- or two-sided<br>*Only common tests should be described solely by name; describe more complex techniques in the Methods section.*
☐	☒	A description of all covariates tested
☐	☒	A description of any assumptions or corrections, such as tests of normality and adjustment for multiple comparisons
☐	☒	A full description of the statistical parameters including central tendency (e.g. means) or other basic estimates (e.g. regression coefficient) AND variation (e.g. standard deviation) or associated estimates of uncertainty (e.g. confidence intervals)
☐	☒	For null hypothesis testing, the test statistic (e.g. *F*, *t*, *r*) with confidence intervals, effect sizes, degrees of freedom and *P* value noted<br>*Give P values as exact values whenever suitable.*
☒	☐	For Bayesian analysis, information on the choice of priors and Markov chain Monte Carlo settings
☐	☒	For hierarchical and complex designs, identification of the appropriate level for tests and full reporting of outcomes
☐	☒	Estimates of effect sizes (e.g. Cohen's *d*, Pearson's *r*), indicating how they were calculated

Our web collection on statistics for biologists contains articles on many of the points above.

Software and code

Policy information about availability of computer code

Data collection	Microscopy images were taken by a digital inverted microscope (EVOS FL) using EVOS AMF4300 imaging system.<br>H&E staining images were taken by an Aperio Scanscope (CS2) using ISCapture 3.9 software.<br>Stereo microscope images were taken by a stereomicroscope (Olympus SZ61) using ISCapture 3.9 software.<br>MATLAB scripts for running SHARP are freely available for download on GitHub (https://github.com/alexanderuernst/SHARP) (license for COMSOL with MATLAB needed).<br>Scripts for training the machine learning model are freely available for download on GitHub (https://github.com/scworland/sharp-ml).<br>Among the constituent models, lightGBM (v3.3.2), XGBoost (v1.6.0.1), and kernel K nearest neighbors (v1.1.4) were used.<br>SHARP-ML may be accessed at https://worland.shinyapps.io/sharp-ml/ (username: guest, password: sharp-ml).<br>Scripts for the web application can be freely downloaded on GitHub ((https://github.com/scworland/sharp-ml-app).<br>Some schematics in Figs. 1a and 4a were created with BioRender.com
Data analysis	GraphPad Prism 8 software were used for data plotting.<br>R 4.1.1 software and GraphPad Prism 8 software were used for statistical analyses.<br>COMSOL Multiphysics 5.4 software with MATLAB software 2019a was used for computational modeling.<br>ImageJ 1.52p software was used for image processing.

For manuscripts utilizing custom algorithms or software that are central to the research but not yet described in published literature, software must be made available to editors and reviewers. We strongly encourage code deposition in a community repository (e.g. GitHub). See the Nature Research guidelines for submitting code & software for further information.

Data

Policy information about availability of data

All manuscripts must include a data availability statement. This statement should provide the following information, where applicable:

- Accession codes, unique identifiers, or web links for publicly available datasets
- A list of figures that have associated raw data
- A description of any restrictions on data availability

Data availability: All data supporting the findings of this study are available within the article and the supplementary information files and from the corresponding author upon reasonable request. Data collected from the Alberta Diabetes Institute IsletCore at the University of Alberta can be found by following registration instructions at https://www.epicore.ualberta.ca/isletcore/Default. Source data are provided with this paper.

Field-specific reporting

Please select the one below that is the best fit for your research. If you are not sure, read the appropriate sections before making your selection.

☒ Life sciences ☐ Behavioural & social sciences ☐ Ecological, evolutionary & environmental sciences

For a reference copy of the document with all sections, see nature.com/documents/nr-reporting-summary-flat.pdf

Life sciences study design

All studies must disclose on these points even when the disclosure is negative.

Sample size	All sample sizes are indicated in the figure legends, and the sample sizes were sufficient to conduct reasonable statistical analyses where applicable. Sample size was selected a priori by estimating the expected difference in therapeutic potency between the two groups using the computational model, and running statistical analysis on the resultant (stochastic) data (see Figs 4f-h), which determined that the sample size was likely sufficient.
Data exclusions	No data was excluded.
Replication	All code is open source to aid reproducibility of computational analysis. In vitro/in vivo data supported computational conclusions.<br>The in vivo experiment was not repeated.
Randomization	For mouse studies, mice were randomly allocated by body weight and level of diabetic state (blood glucose level after diabetes induction) to the experimental groups.<br>All simulations (i.e., all other analyses) were inherently randomized with the Matlab seed randomized each time before running the simulation.
Blinding	No formal blinding was used. However, the blood glucose monitoring was performed by different individuals (though unblinded, as both individuals eligible and available for blood glucose measurements for the duration of the implant also performed the surgeries and thus recognized the cage IDs) and the GSIS ELISA measurement was performed in a blind way.<br>Blinding was not relevant for simulations.

Reporting for specific materials, systems and methods

We require information from authors about some types of materials, experimental systems and methods used in many studies. Here, indicate whether each material, system or method listed is relevant to your study. If you are not sure if a list item applies to your research, read the appropriate section before selecting a response.

Materials & experimental systems

n/a	Involved in the study	
☒	☐	Antibodies
☒	☐	Eukaryotic cell lines
☒	☐	Palaeontology and archaeology
☐	☒	Animals and other organisms
☒	☐	Human research participants
☒	☐	Clinical data
☒	☐	Dual use research of concern

Methods

n/a	Involved in the study	
☒	☐	ChIP-seq
☒	☐	Flow cytometry
☒	☐	MRI-based neuroimaging

Animals and other organisms

Policy information about studies involving animals; ARRIVE guidelines recommended for reporting animal research

Laboratory animals	8-week-old male C57BL/6J mice (Stock No: 000664) were purchased from the Jackson Laboratory (Bar Harbor, ME).<br>The mice were maintained at a temperature of 70-72°F with 30-70% humidity under a 14-hour light/10-hour dark cycle.<br>8-week-old male Sprague-Dawley rats (Strain Code 400, weight ~300 g) were purchased from Charles River Laboratories (Wilmington, MA).<br>The rats were maintained at a temperature of 70-72°F with 30-70% humidity under a 12-hour dark/12-hour light cycle.<br>All research complied with relevant ethical regulations and were approved by the Cornell Institutional Animal Care and Use Committee (Protocol #2012-0144).
Wild animals	No studies involved wild animals
Field-collected samples	No studies involved samples collected from the field.
Ethics oversight	The Cornell Institutional Animal Care and Use Committee approved all animal procedures.

Note that full information on the approval of the study protocol must also be provided in the manuscript.
